# On Edge Exchangeable Random Graphs

**DOI:** 10.1007/s10955-017-1832-9

**Published:** 2017-06-30

**Authors:** Svante Janson

**Affiliations:** Department of Mathematics, Uppsala University, PO Box 480, 751 06 Uppsala, Sweden

**Keywords:** Edge exchangeable random graphs, Graphons, Dense and sparse graph limits, 05C80, 05C65

## Abstract

We study a recent model for edge exchangeable random graphs introduced by Crane and Dempsey; in particular we study asymptotic properties of the random simple graph obtained by merging multiple edges. We study a number of examples, and show that the model can produce dense, sparse and extremely sparse random graphs. One example yields a power-law degree distribution. We give some examples where the random graph is dense and converges a.s. in the sense of graph limit theory, but also an example where a.s. every graph limit is the limit of some subsequence. Another example is sparse and yields convergence to a non-integrable generalized graphon defined on $(0,\infty )$.

## Introduction

A model for edge exchangeable random graphs and hypergraphs was recently introduced by [11, 12], who also gave a representation theorem showing that every infinite edge exchangeable random hypergraph can be constructed by this model. An equivalent model, using somewhat different formulations, was given by [7, 8], see Remark 4.7.

The idea of the model is that random i.i.d. edges, with an arbitrary distribution, are added to a fixed vertex set; see Sect. 4 for a detailed definition (slightly modified but equivalent to the original definition).

The general model defines a random hypergraph. In the present paper, we concentrate on the graph case, although we state the definitions in Sect. 4 more generally for hypergraphs.

Since edges can be repeated, the model defines a random multigraph, but this can as always be reduced to a random simple graph by identifying parallel edges and deleting loops. Typically, many of the edges will be repeated many times, see e.g. Remark 6.7, and thus the multigraph and the simple graph versions can be expected to be quite different. Both versions have interest and potential, possibly different, applications, and we consider both versions. Previous papers concentrate on the multigraph version; in contrast and as a complement, in the present paper we study mainly the simple graph version.

The model is, as said above, based on an arbitrary distribution of edges. Different choices of this distribution can give a wide range of different types of random graphs, and the main purpose of the paper is to investigate the types of random graphs that may be created by this model; for this purpose we give some general results on the numbers of vertices and edges, and a number of examples ranging from dense to very sparse graphs. The examples show that the model can produce very different graphs. In some dense examples we show that the random graphs converge in the sense of graph limit theory. However, that is not always the case, and we even give a chameleon example (Theorem 8.7) that has every graph limit as the limit of some subsequence. We give also a sparse example (Example 9.1) with a power-law degree distribution and convergence to a generalized graphon in the sense of [40].

An important tool in our investigations is a Poisson version of the construction by [12], see Sect. 4.2, which seems interesting also in its own right.

After some preliminaries in Sects. 2–3, we give the definitions of the random hypergraphs in detail in Sect. 4. The graph case is discussed further in Sect. 5. Section 6 studies the numbers of vertices and edges in the graphs. Section 7 considers an important special case of the model, called *rank 1*; we study two multigraph examples previously considered by [11, 36] and show that they are of this type.

The remaining sections consider various examples of the simple graph version, with dense examples in Sect. 8, and sparse examples in Sects. 9 and 10. Finally, we give some tentative conclusions in Sect. 11.

## Some Notation

In general, we allow hypergraphs to have multiple edges; we sometimes (but usually not) say multihypergraph for emphasis. Moreover, the edges in a hypergraph may have repeated vertices, i.e., the edges are in general multisets of vertices, see Remark 4.3. An edge with repeated vertices is called a loop. A simple hypergraph is a hypergraph without multiple edges and loops. (*Warning*: different authors give different meanings to “simple hypergraph”.)

The vertex and edge sets of a multigraph *G* are denoted by *V*(*G*) and *E*(*G*), and the numbers of vertices and edges by $v(G):=|V(G)|$ and $e(G):=|E(G)|$.

$f(x)\sim g(x)$ means $f(x)/g(x)\rightarrow 1$ (as *x* tends to some limit, e.g. $x\rightarrow \infty$). We also use $v\sim w$ for adjacency of two vertices *v* and *w* in a given graph, and $X\sim {\mathcal L}$ meaning that the random variable *X* has distribution ${\mathcal L}$; there should not be any risk of confusion between these (all standard) uses of $\sim$.

$f(x)\asymp g(x)$ for two non-negative functions or sequences *f*(*x*) and *g*(*x*) (defined on some common set *S*) means that *f* / *g* and *g* / *f* both are bounded; equivalently, there exist constants $c,C>0$ such that $cg(x)\leqslant f(x)\leqslant C g(x)$ for every $x\in S$. $f(x)\asymp g(x)$ as ${x\rightarrow \infty }$means that $f(x)\asymp g(x)$ for *x* in some interval $[x_0,\infty )$.

We use ‘increasing’ (for a function or a sequence) in its weak sense i.e., $x\leqslant y\implies f(x)\leqslant f(y)$, and similarly with ’decreasing’.

$x\wedge y$ is $\min \{x,y\}$ and $x\vee y$ is $\max \{x,y\}$.

$$\mathbb N:=\{1,2,\dots \}$$ and $$\mathbb N_0:=\{0,1,2,\dots \}$$. $$[n]:=\{1,\dots ,n\}$$.

If $\mu$ is a measure on a set ${\mathcal S}$, then $\Vert \mu \Vert :=\mu ({\mathcal S})\leqslant \infty$.

${\text {Exp}}(\lambda )$ denotes the exponential distribution with *rate* $\lambda$, i.e., the first point in a Poisson process with rate $\lambda$; this is thus the exponential distribution with mean $1/\lambda$. For convenience we extend this to $\lambda =0$: $X\sim {\text {Exp}}(0)$ means $X=+\infty$ a.s.

We say that a sequence $G_n$ of simple graphs with $v(G_n)\rightarrow \infty$ is *dense* if $e(G_n)\asymp v(G_n)^2$, *sparse* if $e(G_n)=o(v(G_n)^2)$, and *extremely sparse* if $e(G_n)\asymp v(G_n)$ as ${n\rightarrow \infty }$, and similarly for a family $G_t$ of graphs with a continuous parameter.

We let $C,c,C_1,c_1,\dots$ denote various unspecified positive constants.

## Some Preliminaries on Graph Limits, Graphons and Cut Metric

We recall some basic facts on graph limits and graphons. For further details, see e.g. [5, 6, 14] and the comprehensive book [32].

A (standard) *graphon* is a symmetric measurable function $W:\Omega \times \Omega \rightarrow [0,1]$, where $\Omega =(\Omega ,\mathcal F,\mu )$ is a probability space. ($\Omega$ may without loss of generality be taken as $[0,1]$ with Lebesgue measure, but it is sometimes convenient to use other probability spaces too.)

If $\varphi :\Omega _1\rightarrow \Omega _2$ is a measure-preservingmap between two probability spaces $\Omega _1$ and $\Omega _2$, and *W* is a graphon on $\Omega _2$, then $W^\varphi (x,y):=W(\varphi (x),\varphi (y))$ is a graphon on $\Omega _1$ called the *pull-back* of *W*.

If *W* is an integrable function on $\Omega ^2$, then its *cut norm* is

3.1 $$\begin{aligned} \Vert W\Vert _{\square }:=\sup \Bigl |\int _{T\times U}W(x,y)\,\mathrm {d}\mu (x)\,\mathrm {d}\mu (y)\Bigr |, \end{aligned}$$

taking the supremum over all measurable sets $T,U\subseteq \Omega$.

For two graphons $W_1$ and $W_2$, defined on probability spaces $\Omega _1$ and $\Omega _2$, their *cut distance* is defined as

3.2 $$\begin{aligned} \delta _{\square }(W_1,W_2) = \inf _{\varphi _1,\varphi _2}\Vert W_1^{\varphi _1}-W_2^{\varphi _2}\Vert _{\square }, \end{aligned}$$

taking the infimum over all pairs $(\varphi _1,\varphi _2)$ of measure-preservingmaps $\varphi _j:\Omega \rightarrow \Omega _j$ defined on some common probability space $\Omega$.

Two graphons $W_1$ and $W_2$ are *equivalent* if $\delta _{\square }(W_1,W_2)=0$. Note that a graphon *W* and any pullback $W^{\varphi }$ of it are equivalent. For characterizations of equivalent graphons, see [4] and [22, Sect. 8]. The cut distance $\delta _{\square }$ can be regarded as a metric on the set $\mathcal W$ of equivalence classes of graphons, and makes $\mathcal W$ into a compact metric space.

A *graph limit* can be identified with an equivalence class of graphons, so we can regard $\mathcal W$ as the space of graph limits. Thus, every graphon defines a graph limit, and every graph limit is represented by some graphon, but this graphon is unique only up to equivalence.

For every finite graph *G*, there is a corresponding graphon $W_G$ that can be defined by taking $\Omega =V(G)$ with the uniform probability measure $\mu \{i\}=1/v(G)$ for every $i\in V(G)$ and letting $W_G(i,j):=\varvec{1}_{\{i\sim j\}}$; thus $W_G$ equals the adjacency matrix of *G*, regarded as a function $V(G)^2\rightarrow \{0,1\}$. ($W_G$ is often defined as an equivalent graphon on $[0,1]$; for us this makes no difference.) We identify *G* and $W_G$ when convenient, and write for example $\delta _{\square }(G,W)=\delta _{\square }(W_G,W)$ for a graph *G* and a graphon *W*.^1^

### Remark 3.1

Let *G* be a finite graph. A *blow-up* $G^*$ of *G* is the graph obtained by taking, for some integer $m\geqslant 1$, the vertex set $V(G^*)=V(G)\times [m]$ with $(v,i)\sim (w,j)$ in $G^*$ if and only if $v\sim w$ in *G*. Then, $W_{G^*}$ is a pull-back of $W_G$ (for $\varphi :V(G^*)\rightarrow V(G)$ the natural projection), and thus $\delta _{\square }(G^*,G)=\delta _{\square }(W_G,W_{G^*})=0$. Hence the graphs *G* and $G^*$, which are different (if $m>1$) are equivalent when regarded as graphons.

There are several, quite different but nevertheless equivalent, ways to define convergence of a sequence of graphs, see e.g. [5, 6, 14, 32]. For our purposes it suffices to know that a sequence $G_n$ with $v(G_n)\rightarrow \infty$ is *convergent* if and only if there exists a graphon *W* such that $\delta _{\square }(G_n,W)\rightarrow 0$ as ${n\rightarrow \infty }$. We then say that $G_n$ converges to *W*, or to the corresponding graph limit.

### Remark 3.2

The standard graphons defined above are appropriate for dense graphs. For sparse graphs, other, more general, graphons have been constructed by several authors. We will in Sect. 5.1 compare the edge exchangeable graphs studied in the present paper with random graphs defined by graphons that are defined on $\mathbb R_+$ or another infinite ($\sigma$-finite) measure space instead of a probability space, see [39, 3]. Furthermore, in Sect. 9 we consider an example of edge exchangeable graphs that yields sparse graphs, where we show that the graphs converge in a suitable sense (see [40]) to such a graphon defined on $\mathbb R_+$. We postpone the definitions to these sections.

## Constructions of Random Hypergraphs

In this section, we define the random hypergraphs. We give several versions; we define both multihypergraphs and simple hypergraphs, and we give both the original version with a fixed number of edges and a Poisson version. In later sections we consider only the graph case, but we give the definitions here in greater generality.

Note that the edge exchangeable random hypergraphs constructed here are quite different from the vertex exchangeable graphs in e.g. [5, 6, 32, 9, 39, 3], see Sect. 5.1.

We begin with some preliminaries.

Let $({\mathcal S},\mathcal F)$ be a measurable space, for convenience usually denoted simply by ${\mathcal S}$. To avoid uninteresting technical complications, we assume that ${\mathcal S}$ is a Borel space, i.e., isomorphic to a Borel subset of a complete separable metric space with its Borel $\sigma$-field.

Let ${\mathcal S}^*$ be the set of all finite non-empty multisets of points in ${\mathcal S}$. We can regard a multiset with *n* elements as an equivalence class of sequences $(x_1,\dots ,x_n)\in {\mathcal S}^n$, where two such sequences are equivalent if one is a permutation of the other. Denoting this equivalence relation by $\cong$ and the set of multisets of *n* elements in ${\mathcal S}$ by ${\mathcal S}^{\vee n}$, we thus have ${\mathcal S}^{\vee n}={\mathcal S}^n/{\cong }$ and ${\mathcal S}^*=\bigcup _{n=1}^\infty {\mathcal S}^{\vee n}$. Note that ${\mathcal S}^{\vee n}$ and ${\mathcal S}^*$ are Borel spaces. (One way to see this is to recall that every Borel space is isomorphic to a Borel subset of $[0,1]$. We may thus assume that ${\mathcal S}\subseteq [0,1]$, and then we can redefine ${\mathcal S}^{\vee n}$ as $\{(x_1,\dots ,x_n)\in {\mathcal S}^n:x_1\leqslant \dots \leqslant x_n\}$, which is a Borel subset of $[0,1]^n$.)

### Remark 4.1

Definitions 4.2 and 4.8 below use a probability measure $\mu$ to define the random (hyper)graphs. In general, this measure may be a random measure, and then the constructions should be interpreted by conditioning on $\mu$, i.e., by first sampling $\mu$, and then using the obtained measure throughout the construction. In other words, the distribution of the random hypergraphs constructed by a random measure $\mu$ is a mixture of the distributions given by deterministic $\mu$. For convenience, and because most examples will be with deterministic $\mu$, we usually tacitly assume that $\mu$ is deterministic; results in the general case with random $\mu$ then follow by conditioning on $\mu$. (See Remark 4.11 for a typical example, where this for once is stated explicitly.)

### Random Hypergraphs with a Given Number of Edges

We give a minor modification of the original definition by [11, 12]; we will see at the end of this subsection that our definition is equivalent to the original one.

#### Definition 4.2

Given a Borel space ${\mathcal S}$ and a probability measure $\mu$ on ${\mathcal S}^*$, define a sequence of finite random (multi)hypergraphs $(G^*_m)_{m=1}^\infty$ as follows.

4.1 $$\begin{aligned} \begin{array}{ll} \text {(i)}\,&{}\quad \text {Draw }\, Y_1,Y_2,\dots \sim _{\mathrm {iid}}\mu . \\ \text {(ii)}&{}\quad \text {Let }\, V(G^*_m):=\bigcup _{i\leqslant m} Y_i\, \text { and }\, E(G^*_m):=\{Y_1,\dots ,Y_m\} \text { (multiset).} \end{array} \end{aligned}$$

Note that $V(G^*_m)$ is the vertex set spanned by the edges; thus there are no isolated vertices in $G^*_m$. (The same holds for the related definitions in (4.2), (4.6), (4.7) below.)

We also similarly define the infinite (multi)hypergraph $G^*_\infty$ having edges $(Y_i)_{i=1}^\infty$.

The edges in $G^*_m$ may be repeated, so $G^*_m$ is in general a random multihypergraph. We define $G_m$ as the simple hypergraph obtained by merging each set of parallel edges in $G^*_m$ to a single edge and deleting loops; thus the simple hypergraphs $(G_m)_1^\infty$ are defined by:

4.2 $$\begin{aligned} \begin{array}{ll} \text {(i)}&{}\quad \text {Draw }\, Y_1,Y_2,\dots \sim _{\mathrm {iid}}\mu . \\ \text {(ii)}&{}\quad \text {Let }\, E(G_m)=\{Y_i:i\leqslant m,\,Y_i\,\text { not loop}\}\, \text { and }\, \\ &{}\quad V(G_m)=\bigcup _{Y_i\in E(G_m)} Y_i. \end{array} \end{aligned}$$

Thus $V(G_m)\subseteq V(G^*_m)$, and strict inequality is possible if there are loops.

Note that $G^*_1\subset G^*_2\subset \cdots$, and thus $G_1\subseteq G_2\subseteq \cdots$, i.e., $(G^*_m)_m$ and $(G_m)_m$ are increasing sequences of random hypergraphs.

#### Remark 4.3

We follow [12] and allow for increased generality $$Y_i$$ to be a multiset (see e.g. the examples in Sect. 7); thus the edges in $$G^*_m$$ and $$G_m$$ are multisets and may contain repeated vertices. If we choose $$\mu $$ with support in the set $${\mathcal S}^{**}:=\bigcup _{n=1}^\infty {\mathcal S}^{\wedge n}\subset {\mathcal S}^*$$ of finite subsets of $${\mathcal S}$$, where $${\mathcal S}^{\wedge n}\subset {\mathcal S}^{\vee n}$$ is the set of subsets of $${\mathcal S}$$ with *n* distinct elements, then the edges in $$G^*_m$$ and $$G_m$$ are ordinary sets of vertices (i.e., without repeated vertices). (This is commonly assumed in the definition of hypergraphs.)

In particular, if $\mu$ has support in ${\mathcal S}^{\wedge 2}=\{\{x,y\}:x,y\in {\mathcal S},\,x\ne y\}$, then $G^*_m$ is a multigraph without loops, and $G_m$ is a simple graph with $V(G_m)=V(G^*_m)$.

The construction above yields hypergraphs with vertices labelled by elements of ${\mathcal S}$. We (usually) ignore these labels and regard $G^*_m$ and $G_m$ as unlabelled hypergraphs.

#### Remark 4.4

We usually also ignore the labels on the edges. If we keep the labels *i* on the edges $Y_i$, then the distribution of $G^*_m$ is obviously edge exchangeable, i.e., invariant under permutations of these edge labels, because $(Y_i)_i$ is an i.i.d. sequence. Conversely, as shown by [12, Theorem 3.4], every infinite edge exchangeable hypergraph is a mixture of random hypergraphs $G^*_\infty$, i.e., it can be constructed as above using a random measure $\mu$. In the present formulation, the proof in [12] simplifies somewhat: give the vertices in the edge exchangeable hypergraph random labels that are i.i.d. and *U*(0, 1) (uniformly distributed on $[0,1]$), and independent of the edges. Then the edges become multisets in $[0,1]^*$, and their distribution is clearly exchangeable, so by de Finetti’s theorem, the edges are given by the construction above for some random probability measure $\mu$ on ${\mathcal S}^*$, taking ${\mathcal S}=[0,1]$.

It is obvious from the definition that if $\psi :{\mathcal S}\rightarrow {\mathcal S}_1$ is an injective measurable map of ${\mathcal S}$ into another measurable (Borel) space ${\mathcal S}_1$, then $\mu$ is mapped to a probability measure $\mu _1$ on ${\mathcal S}^*_1$, which defines the same random hypergraphs $G^*_m$ and $G_m$ as $\mu$. Hence, the choice of Borel space ${\mathcal S}$ is not important, and we can always use e.g. ${\mathcal S}=[0,1]$. Moreover, we can simplify further.

Define the *intensity* of $\mu$ as the measure on $({\mathcal S},\mathcal F)$

4.3 $$\begin{aligned} \bar{\mu }(A):={\mathbb {E}}|A\cap Y|, \qquad A\in \mathcal F, \end{aligned}$$

where *Y* has distribution $\mu$. Note that for a singleton set $\{x\}$, $|\{x\}\cap Y|=\varvec{1}_{\{x\in Y\}}$, and thus (4.3) yields

4.4 $$\begin{aligned} \bar{\mu }(\{x\})={\mathbb {P}}(x\in Y). \end{aligned}$$

We have $\bar{\mu }(A)=\sum _{n=1}^\infty \bar{\mu }_n(A)$, where $\bar{\mu }_n(A):={\mathbb {E}}\bigl (|A\cap Y|\cdot \varvec{1}_{\{|Y|=n\}}\bigr )$, and since each $\bar{\mu }_n$ is a finite measure, it follows that the set of atoms

4.5 $$\begin{aligned} \mathcal A:=\{x\in {\mathcal S}:\bar{\mu }(\{x\})>0\} \end{aligned}$$

is a countable (finite or infinite) subset of ${\mathcal S}$. By (4.4) and (4.5), if $x\notin \mathcal A$, then ${\mathbb {P}}(x\in Y)=0$. Hence, in the construction of $G^*_m$, if an edge $Y_i$ has a vertex $x\notin \mathcal A$, then a.s $x\notin Y_j$ for every $j\ne i$. Consequently, a vertex $x\notin \mathcal A$ of $G^*_\infty$ a.s appears in only one edge. (Such a vertex is called a *blip* in [12].) On the other hand, if $x\in \mathcal A$, so ${\mathbb {P}}(x\in Y)=\bar{\mu }(\{x\})>0$, then by the law of large numbers, a.s *x* belongs to infinitely many edges $Y_i$ of $G^*_\infty$.

It follows that when constructing the hypergraphs $G^*_m$, if the edge $Y_i=\{y_{i1},\dots ,y_{in_i}\}$, we do not have to keep track of the vertex labels $y_{ij}$ unless they belong to $\mathcal A$; any $y_{ij}\notin \mathcal A$ will be a blip not contained in any other edge and the actual value of $y_{ij}$ may be forgotten. (Except that if we allow repeated vertices in the edges, see Remark 4.3, then we still have to know whether two vertex labels $y_{ij}$ and $y_{ik}$ on the same edge are the same or not.)

Now, enumerate $\mathcal A$ as $\{a_i\}_{i=1}^N$, where $N\leqslant \infty$, and replace, for every multiset $Y=(y_1,\dots ,y_\ell )\in {\mathcal S}^*$, every vertex label $y_{j}=a_k$ for some $a_k\in \mathcal A$ by the new label $y'_j=k$, and the vertex labels $y_{j}\notin \mathcal A$ on *Y* by $0, -1, \dots$. (For definiteness, we may assume that ${\mathcal S}\subseteq [0,1]$ so ${\mathcal S}$ is ordered, and take the labels in order in case *Y* has more than one vertex label not in $\mathcal A$.) This maps $\mu$ to a probability measure $\mu '$ on the set $\mathbb Z^*$ of finite multisets of integers, and it follows from the discussion above that we can recover the random hypergraphs $G^*_m$ from $\mu '$ by the construction in Definition 4.2, if we first replace each vertex label $y_j'\in \{0,-1,\dots \}$ by a random label with a continuous distribution in some set, for example *U*(0, 1), making independent choices for each $Y_i$. Equivalently, and more directly, we obtain $G^*_m$ from the probability measure $\mu '$ on $\mathbb Z^*$ by the following construction, which is the original definition by [11, 12].

#### Definition 4.5

[11, 12] Given a probability measure $\mu$ on $\mathbb Z^*$, we define a sequence of finite random (multi)hypergraphs $(G^*_m)_{m=1}^\infty$ as in Definition 4.2 with the modification that in every edge $Y_i=\{y_{i1},\dots ,y_{i\ell _i}\}$ we replace every vertex label $y_{ij}\leqslant 0$ (if any) with a new vertex that is not used for any other edge.

Since we ignore the vertex labels in $G^*_m$, it does not matter what labels we use as replacements for $0,-1,\dots$ in Definition 4.5. Crane and Dempsey [11, 12] use the same set $0,-1,\dots$ of integers, taking the first label not already used. An alternative is to take random labels, e.g. i.i.d. *U*(0, 1) as above.

#### Remark 4.6

To be precise, Definition 4.5 is the definition in [12]. The definition in [11] treats only the binary case $|Y_n|=2$ in detail; and differs in that only labels $y_i\geqslant 0$ are used, and that an edge $\{0,0\}$ is replaced by an edge $\{z_1,z_2\}$ with two new vertex labels $z_1$ and $z_2$.

This version is essentially equivalent; apart from a minor notational difference, the only difference is that this version does not allow for “loop dust”, where a positive fraction of the edges are isolated loops. Cf. Remark 5.2.

We have shown that Definition 4.2 is essentially equivalent to the original definitions by [11, 12]. One advantage of Definition 4.2 is that no special treatment of vertex labels $\leqslant 0$ is needed; the blips (if there are any) come automatically from the continuous part of the label distribution; a disadvantage is that this continuous part is arbitrary and thus does not contain any information. Another advantage with Definition 4.2 is that it allows for arbitrary Borel spaces ${\mathcal S}$; even if it usually is convenient to use ${\mathcal S}=\mathbb N$ to label the vertices, it may in some examples be natural to use another set ${\mathcal S}$.

#### Remark 4.7

The construction in [8] is stated differently, but is equivalent. It uses a generalization of Kingman’s paintbox construction of exchangeable partitions; in the version in [8], the paintbox consists of families $(C_{kj})_{k,j\geqslant 1}$ and $(C'_{jl})_{j,l\geqslant 1}$ of subsets of $[0,1]$; it is assumed that every $x\in [0,1]$ is an element of only finitely many of these sets, and that for each *j* and $k\ne l$, $C_{jk}\cap C_{jl}=\emptyset$ and $C'_{jk}\cap C'_{jl}=\emptyset$. (In general these sets may be random, but similarly as above, in the construction we condition on these sets so we may assume that they are deterministic.) Furthermore, we generate i.i.d. *U*(0, 1) random labels $\phi _k$ and $\phi _{Njl}$ for $k,N,j,l\geqslant 1$. For each $N\geqslant 1$ we construct a edge $Y_N$ by taking a uniformly random point $V_N\in [0,1]$, independent of everything else; then, for each (*j*, *k*) such that $V_N\in C_{jk}$, $Y_N$ contains *k* vertices labelled $\phi _j$, and for each (*j*, *k*) such that $V_N\in C'_{jk}$ and every $l\leqslant k$, $Y_N$ contains *j* vertices labelled $\phi _{Njl}$. (The latter vertices are thus blips.)

Note that this gives the vertices random labels as in Remark 4.4; however, we then ignore the vertex labels. (Actually, in [8], each vertex is represented by a multiset of edge labels (called a *trait*), which contains the label of each edge that contains the vertex, repeated as many times as the vertex occurs in the edge. This is obviously an equivalent way to describe the hypergraph.)

It is obvious that, conditioned on the labels $\phi _k$ and $\phi _{Njl}$, this construction gives a random multiset with some distribution $\mu$; conversely, every distribution $\mu$ of a random (finite) multiset can easily be obtained in this way by suitable choices of $C_{jk}$ and $C'_{jk}$. Hence, the construction is equivalent to the one above. (In our opinion, it is more natural to focus on the distribution of the edges, since the sets $C_{jk}$ and $C'_{jk}$ in the paintbox construction have no intrinsic meaning; they are just used to describe the edge distribution.)

### The Poisson Version

The multihypergraph $G^*_m$ has exactly *m* edges (not necessarily distinct). It is often convenient to instead consider a Poisson number. (This was done by Broderick and Cai in [7, Example 2.7].) It is then natural to consider a continuous-parameter family of hypergraphs, which we define as follows. We may think of the second coordinate *t* as time.

#### Definition 4.8

Given a probability measure $\mu$ on ${\mathcal S}^*$, we define a family of random (multi)hypergraphs $(\tilde{G}^*_t)_{t\geqslant 0}$ as follows. Recall that a Poisson point process on an infinite, $\sigma$-finite measure space is a random countably infinite set of points that can be enumerated as in (i), in our case, for some random $Y_i\in {\mathcal S}^*$ and $\tau _i\in [0,\infty )$.

4.6 $$\begin{aligned} \begin{array}{ll} \text {(i)}\quad &{} \text {Let}\, \Xi =\{(Y_i,\tau _i):i\geqslant 1\}\, \text {be a Poisson point process on}\, {\mathcal S}^*\times [0,\infty )\\ &{} \text {with intensity}\, \mu \times \,\mathrm {d}t; \\ \text {(ii)}\quad &{} \text {Let}\, E(\tilde{G}^*_t):=\{Y_i:\tau _i\leqslant t\}\, \text {(multiset),}\, \text {and}\, V(\tilde{G}^*_t):=\bigcup _{Y\in E(\tilde{G}^*_t)} Y. \end{array} \end{aligned}$$

Define $\tilde{G}_t$ as the simple hypergraph obtained by merging each set of parallel edges in $\tilde{G}^*_t$ to a single edge, and deleting loops (together with their incident vertices, unless these also belong to some non-loop). Hence, with (i) as in (4.6),

4.7 $$\begin{aligned} \text {(ii)}\quad&\text {Let}\, E(\tilde{G}_t):=\{Y_i \,\text { not loop}\,:\tau _i\leqslant t\} \,\text {and}\, V(\tilde{G}_t):=\bigcup _{Y\in E(\tilde{G}_t)} Y. \end{aligned}$$

Note that the random hypergraphs $\tilde{G}^*_t$ and $\tilde{G}_t$ are a.s finite for every $t<\infty$.

The projection $\Xi '':=\{\tau _i\}_{i=1}^\infty$ of the Poisson process $\Xi$ to the second coordinate is a Poisson point process on $[0,\infty )$ with intensity 1, and we may and will assume that the points of $\Xi$ are enumerated with $\tau _i$ in increasing order; thus a.s $0<\tau _1<\tau _2<\dots$. Let *N*(*t*) be the number of points of $\Xi$ in ${\mathcal S}^*\times [0,t]$, i.e.

4.8 $$\begin{aligned} N(t):=\bigl |\Xi \cap ({\mathcal S}^*\times [0,t])\bigr |=\max \{i:\tau _i\leqslant t\}; \end{aligned}$$

this is a Poisson counting process on $[0,\infty )$ and $N(t)\sim {\text {Po}}(t)$. Conversely, $\tau _m$ is the time the process *N*(*t*) reaches *m*, so the increments $\tau _m-\tau _{m-1}$ (with $\tau _0:=0$) are i.i.d. and ${\text {Exp}}(1)$, and $\tau _m$ has the Gamma distribution $\Gamma (m)$. Moreover, the random multisets $Y_i$ are i.i.d. with distribution $\mu$ and independent of $\{\tau _i\}$, so they can be taken as the $Y_i$ in Definition 4.2, which leads to the following simple relation between the two definitions.

#### Proposition 4.9

If $\mu$ is a probability measure on ${\mathcal S}^*$, then we may couple the random hypergraphs constructed in Definitions 4.2 and 4.8 such that $G^*_m=\tilde{G}^*_{\tau _m}$ and thus $G_m=\tilde{G}_{\tau _m}$ for all $m\geqslant 1$, and conversely $\tilde{G}^*_t=G^*_{N(t)}$ and $\tilde{G}_t=G_{N(t)}$ for all $t\geqslant 0$. $\square$

Although we usually tacitly consider $t<\infty$, we may here also take $t=\infty$: $G^*_\infty =\tilde{G}^*_\infty$ and $G_\infty =\tilde{G}_\infty$.

Note that the relations in Proposition 4.9 hold not just for a single *m* or *t*, but also for the entire processes. Hence, asymptotic results, and in particular a.s limit results, are (typically) easily transfered from one setting to the other.

#### Remark 4.10

Instead of stopping at the random time $\tau _m$, we can also obtain $G^*_m$ and $G_m$ from $\tilde{G}^*_t$ and $\tilde{G}_t$ by conditioning on $N(t)=m$, for any fixed $t>0$.

#### Remark 4.11

One reason that the Poisson version is convenient is that different edges appear independently of each other. If we for convenience assume that there are no blips, we may as explained above assume that ${\mathcal S}=\mathbb N$, so $V(\tilde{G}^*_t)\subseteq \mathbb N$. In this case, the number of copies of an edge $I\in {\mathcal S}^*$ in $\tilde{G}^*_t$ has the Poisson distribution ${\text {Po}}(t\mu (\{I\}))$, and these numbers are independent for different $I\in {\mathcal S}^*$. Hence, different edges $I\in {\mathcal S}^*$ appear independently in $\tilde{G}_t$. (In the case $\mu$ is random, this holds conditionally on $\mu$, but not unconditionally.)

Note that this independence does not hold for $G_m$; the stopping in Proposition 4.9 or the conditioning in Remark 4.10 destroys the independence of different edges.

### Unnormalized Measures

We have so far assumed that $\mu$ is a probability measure. This is very natural, but we can make a trivial extension to arbitrary finite measures. This will not produce any new random hypergraphs but it is convenient; for example, it means that we do not have to normalize the measure in the examples in later sections.

When necessary, we denote the measure used in the construction of our random hypergraphs by a subscript; we may thus write e.g. $G_{m,\mu }$.

#### Definition 4.12

Let $\mu$ be a finite measure on ${\mathcal S}^*$, not identically zero. Let $\mu _0$ be the probability measure $\mu _0:=\Vert \mu \Vert ^{-1}\mu$, and define $G^*_{m,\mu }:=G^*_{m,\mu _0}$. Furthermore, define $\tilde{G}^*_{t,\mu }$ as in Definition 4.8. Let, as usual, $G_{m,\mu }$ and $\tilde{G}_{t,\mu }$ be the corresponding simple graphs.

Thus, $\mu =c\mu _0$, where $c:=\Vert \mu \Vert =\mu ({\mathcal S}^*)$. It is obvious that, using obvious notation, the Poisson process $\Xi _\mu$ can be obtained from $\Xi _{\mu _0}$ by rescaling the time: if $\Xi _{\mu _0}=\{(Y_i,\tau ^0_i)\}$, we can take $\Xi _\mu =\{(Y_i,c^{-1}\tau ^0_i)\}$, and thus $G^*_{t,\mu }=G^*_{ct,\mu _0}$. Hence, the random hypergraph process defined by $\mu$ is the same as for $\mu _0$, except for a simple deterministic change of time. This implies the following result.

#### Proposition 4.13

Proposition 4.9 extends to arbitrary finite measures $\mu$ (not identically zero), with stopping times $\tau _m$ that are the partial sums $\sum _{i=1}^mT_i$ of i.i.d. random variables $T_i\sim {\text {Exp}}(\Vert \mu \Vert )$.

In particular, the law of large numbers yields, as ${m\rightarrow \infty }$,

4.9 $$\begin{aligned} \tau _m/m\overset{\mathrm {a.s.}}{\longrightarrow }\Vert \mu \Vert ^{-1}. \end{aligned}$$

#### Remark 4.14

Definition 4.8 can be employed also when $\mu$ is an infinite and, say, $\sigma$-finitemeasure. In this case, $\tilde{G}^*_t$ has a.s an infinite number of edges for every $t>0$. We will not consider this case further.

## Random Graphs

From now on, we consider the graph case, where $\mu$ is a finite measure on ${\mathcal S}^{\vee 2}=\{\{x,y\}:x,y\in {\mathcal S}\}$. This allows for the presence of loops; often we consider $\mu$ supported on ${\mathcal S}^{\wedge 2}=\{\{x,y\}:x\ne y\}$, and then there are no loops.

As explained in Sect. 4, in particular Definition 4.5, if there are no blips (i.e., if the intensity $\bar{\mu }$ is discrete), we may without loss of generality assume that ${\mathcal S}=\mathbb N$, and if there are blips, we may assume that ${\mathcal S}=\mathbb N\cup \{0,-1\}$ with the special convention that 0 and $-1$ are interpreted as blips. Unless stated otherwise, we use this version, and we then write $\mu _{ij}$ for $\mu (\{i,j\})$; we say that $\mu _{ij}$ is the *intensity* of edges *ij*. Thus, $(\mu _{ij})$ is an infinite symmetric matrix of non-negative numbers, with indices in $\mathbb N\cup \{0,-1\}$ (or in $\mathbb N$ if there are no blips); note that, because we consider undirected edges, the total mass of $\mu$ is

5.1 $$\begin{aligned} \Vert \mu \Vert =\frac{1}{2}\sum _{i,j:\;i\ne j}\mu _{ij}+\sum _i \mu _{ii}. \end{aligned}$$

We assume that $0<\Vert \mu \Vert <\infty$, or equivalently that $\sum _{i,j}\mu _{ij}$ is finite (and non-zero), but we do not insist on $\mu$ being a probability measure. As described in Sect. 4.3, we can always normalize $\mu$ to the probability measure $\Vert \mu \Vert ^{-1}\mu$ when desired.

We also define (for $i\geqslant 1$)

5.2 $$\begin{aligned} \mu _i:=\sum _j\mu _{ij}, \end{aligned}$$

this is the total intensity of edges adjacent to vertex *i*.

### Remark 5.1

The diagonal terms $\mu _{ii}$ correspond to loops. Loops appear naturally in some examples, see e.g. Example 7.1 below, but we are often interested in examples without loops, and then take $\mu _{ii}=0$. Moreover, in the construction of the simple graphs $G_m$ and $\tilde{G}_t$ we delete loops, so it is convenient to take $\mu _{ii}=0$ and avoid loops completely. Note that, since different edges appear independently in $\tilde{G}^*_t$, see Remark 4.11, deleting all loops from $\tilde{G}^*_t$ is equivalent to conditioning $\tilde{G}^*_t$ on containing no loops; this is also equivalent to changing every $\mu _{ii}$ to 0. (For $G_m$ this is not quite true, since the number of non-loop edges may change; however, the difference is minor.)

Note also the in the construction leading to Definition 4.5, in the graph case, vertex label $-1$ is used only for the edge $\{0,-1\}$, so we may (and will) assume that $\mu _{i,-1}=0$ unless $i=0$.

Suppose now that we are given such a matrix $(\mu _{ij})_{i,j\geqslant -1}$. We can decompose the matrix into the three parts $(\mu _{ij})_{i,j\geqslant 1}$, $(\mu _{i0})_{i\geqslant 1}$, $(\mu _{i0})_{i\in \{0,-1\}}$, which by the construction and properties of Poisson processes correspond to a decomposition of the Poissonian multigraph $\tilde{G}^*_t$ as a union of three parts, which are independent random graphs:

- Central part: The edges $ij\in \tilde{G}^*_t$ with $i,j\in \mathbb N$.
- Attached stars: For each $i\geqslant 1$ a star with ${\text {Po}}(t\mu _{i0})$ edges centred at *i*.
- Dust: ${\text {Po}}(t\mu _{00})$ isolated loops and ${\text {Po}}(t\mu _{0,-1})$ isolated edges.

Moreover, the Poisson random variables above, for different *i* and for the two types of dust, are independent. The vertex set is by definition the set of endpoints of the edges, so there are no isolated vertices. The edges and loops in the dust are always isolated, i.e.with endpoints that are blips (have no other edges). Similarly, the peripheral vertices in the attached stars are blips without other edges, while the central vertex *i* may, or may not, also belong to the central part.

Note that multiple edges only occur in the central part.

### Remark 5.2

We have here discussed the model in full generality, but it is obvious that the main interest is in the central part, and all our examples will be with $\mu$ supported on $\mathbb N\times \mathbb N$, i.e., without dust and attached stars. (Of course, there may be other stars or isolated edges, created in the central part.)

In particular, the dust part is quite trivial, and the dust loops are even less interesting than the dust edges. In a case with dust but no loops in the dust, it is convenient to relabel $\mu _{0,-1}$ as $\mu _{00}$, so $\mu$ is a symmetric matrix with index set $\mathbb N_0$; this corresponds to using the version of the definition in [11], see Remark 4.6.

### A Comparison with Vertex Exchangeable Graphs

Consider the case without dust, attached stars and loops, so $\mu$ is supported on $\mathbb N\times \mathbb N$, with $\mu _{ii}=0$. Then $\tilde{G}^*_t$ has ${\text {Po}}(t\mu _{ij})$ edges *ij*, for every pair of distinct integers $i,j\in \mathbb N$.

We may compare this to the vertex exchangeable random graphs studied by e.g. [5, 6, 14, 32] and their generalizations by [9, 39, 3], see also [34, 23, 23].

In the classical case [5, 6, 32], with a standard graphon *W* defined on a probability space $(\Omega ,\nu )$ as in Sect. 3, the vertex exchangeable random graph *G*(*n*, *W*) has a given number *n* of vertices and is constructed as follows.

5.3 $$\begin{aligned} \begin{array}{ll} \text {(i)}\quad &{} \text {Draw}\, x_1,x_2,\dots x_n\sim _{\mathrm {iid}}\nu .\,\, (\text {Think of } x_i \text { as the }\, ``\text {type''}\, \text {of}\, i.) \\ \text {(ii)}\quad &{} \text {Let}\, V(G(n,W)):=\{1,\dots ,n\}. \, \text {For each pair}\, (i,j), i<j, \\ &{}\text {let}\, ij\in E(G(n,W))\, \text {with probability}\, W(x_i,x_j). \end{array} \end{aligned}$$

(Different edges are added independently, given the types $x_i$.)

The generalization to graphons on $\mathbb R_+$ or another $\sigma$-finite measure space $(\Omega ,\nu )$ [9, 39, 3] is similar. This type of graphon is still a symmetric measurable function $W:\Omega ^2\rightarrow [0,1]$ (satisfying some conditions to make the graphs *G*(*t*, *W*) defined below a.s finite). $\Omega$ can be regarded as a space of types, and the random graph, here denoted $\bar{G}(t,W)$, is defined as follows. The number of vertices $N\sim {\text {Po}}(t\nu (\Omega ))$ is a random variable, with $N=\infty$ if $\nu$ is an infinite measure.

5.4 $$\begin{aligned}&\text {(i)}\quad \text {Let}\, \Xi =\{x_i\}_{i=1}^N\, \text {be a Poisson point process on}\, \Omega \,\text {with intensity}\, t\nu .\nonumber \\&\text {(ii)}\;\;\text {Let}\, V(\bar{G}(t,W)):=\{i\in \mathbb N:i \leqslant N\}.\, \text {For each pair}\, (i,j), i<j, \nonumber \\&\qquad ~\text {let}\, ij\in E(\bar{G}(t,W)) \,\text {with probability}\, W(x_i,x_j). \end{aligned}$$

Finally, we may delete all isolated vertices, giving a graph *G*(*t*, *W*) without isolated vertices (as in the construction in Sect. 4 above):

5.5 $$\begin{aligned} \begin{array}{ll} \text {(iii)}\quad &{} \text {Let}\, V(G(t,W)):=\{i:ij\in E(\bar{G}(t,W)) \text { for some }\,j\},\\ &{} E(G(t,W)):=E\bigl (\bar{G}(t,W)\bigr ). \end{array} \end{aligned}$$

In both cases (5.3) and (5.4)–(5.5), a natural multigraph version is to modify (ii) by instead taking a Poisson number ${\text {Po}}(W(x_i,x_j))$ of copies of the edge *ij*. (Cf. e.g. [2, Remark 2.4]. One might also take ${\text {Po}}(-\log (1-W(x_i,x_j)))$ copies, keeping the probability of no edge the same as for the Bernoulli model. Note that if the $W(x_i,x_j)$ are small, then the standard (Bernoulli) and the two Poisson versions are almost the same.)

The Poisson versions of the edge exchangeable and vertex exchangeable random graphs thus add edges in the same way, if we condition on the types of the vertices in the latter and let $\mu _{ij}=t^{-1}W(x_i,x_j)$. However, the vertices are constructed in very different ways. To see the similarities and differences clearly, consider the case where the type space $\Omega =\mathbb N$, with some (finite or infinite) measure $\nu$, and consider the Poisson multigraph version of the vertex exchangeable graphs, which we denote by $\bar{G}^*(t,W)$ and $G^*(t,W)$. Then the vertex exchangeable $\bar{G}^*(t,W)$ has a Poisson number ${\text {Po}}(t\nu \{i\})$ of vertices of type *i*, for each $i\in \mathbb N$, while the edge exchangeable $\tilde{G}^*_t$ has at most one vertex *i* for each $i\in \mathbb N$. (We can reformulate the construction of $\tilde{G}^*_t$ and say that we start with exactly one vertex of type *i* for every $i\in \mathbb N$, and then remove all isolated vertices after having added edges.) Moreover, although for a fixed *t*, each pair of distinct vertices of types *i* and *j* has ${\text {Po}}(W(i,j))$ edges between them in $\bar{G}^*(t,W)$ or $G^*(t,W)$ and ${\text {Po}}(t\mu _{ij})$ edges in $\tilde{G}^*_t$, which coincide if $W(i,j)=t\mu _{ij}$, we see that if we keep *W* and $\mu$ fixed and increase *t*, the two families $\bar{G}^*(t,W)$ and $\tilde{G}^*_t$ behave differently: in $\tilde{G}^*_t$ the number of edges between each pair of vertices increases linearly as *t* increases, the number of vertices increases more slowly (by Corollary 6.6 below; recall that we only keep vertices with at least one edge), and there is at most one vertex of each type. In $\bar{G}^*(t,W)$ and $G^*(t,W)$, the number of vertices of each type increases linearly, while the number of edges between each pair of vertices remains the same.

## Numbers of Vertices and Edges

By construction, the number of edges is *m* in the multigraph $G^*_m$ and random ${\text {Po}}(t\Vert \mu \Vert )$ in the multigraph $\tilde{G}^*_t$. The numbers of vertices in the graphs and the numbers of edges in the simple graphs $G_m$ and $\tilde{G}_t$ are somewhat less immediate, and are studied in this section.

We use the notation of Sect. 5, and assume that we are given a (deterministic) matrix $\mu =(\mu _{ij})$ of intensities. Moreover, for simplicity we assume that $\mu$ is concentrated on $\mathbb N\times \mathbb N$, so there are no attached stars and no dust, and that $\mu _{ii}=0$ for every *i*, so there are no loops. We consider briefly the case with dust or attached stars in Sect. 6.1.

Note that $G_m$ is a simple graph without isolated vertices, and thus

6.1 $$\begin{aligned} \tfrac{1}{2} v(G_m)\leqslant e(G_m)\leqslant \left( {\begin{array}{c}v(G_m)\\ 2\end{array}}\right) \leqslant \tfrac{1}{2} v(G_m)^2. \end{aligned}$$

Recall that $G_m$ is *dense* if $e(G_m)\asymp v(G_m)^2$, *sparse* if $e(G_m)=o(v(G_m)^2)$, and *extremely sparse* if $e(G_m)\asymp v(G_m)$ as ${m\rightarrow \infty }$. By Propositions 4.9 and 4.13, these are equivalent to the corresponding conditions for $\tilde{G}_t$.

The number of edges in $G_m$ is the number of different values taken by the i.i.d. sequence $Y_1,\dots ,Y_m$. Equivalently, it is the number of occupied bins if *m* balls are thrown independently into an infinite number of boxes, with the probability $\mu _{ij}$ (normalized if necessary) for box $\{i,j\}$. Such numbers have been studied in, for example, [13, 30, 31, 15, 19], where central limit theorems have been proved under various assumptions, see Theorem 6.8 below. These results are often proved using Poissonization, which in our setting is equivalent to considering $\tilde{G}_t$ instead of $G_m$. We too find it convenient to first study the Poisson version.

The Poisson model is convenient because, as said before, edges *ij* arrive according to a Poisson process with intensity $\mu _{ij}$ and these Poisson processes are independent for different pairs $\{i,j\}$. Let $N_{ij}(t)$ be the number of copies of the edge *ij* in $\tilde{G}^*_t$, and let $N_i(t)$ be the degree of vertex *i* in $\tilde{G}^*_t$. Then

6.2 $$\begin{aligned} N_{ij}(t)\sim {\text {Po}}\bigl (t\mu _{ij}\bigr ), \end{aligned}$$

and, recalling (5.2),

6.3 $$\begin{aligned} N_i(t)=\sum _{j\ne i} N_{ij}(t)\sim {\text {Po}}\bigl (t\mu _i\bigr ). \end{aligned}$$

Moreover, let $T_i\sim {\text {Exp}}(\mu _i)$ and $T_{ij}\sim {\text {Exp}}(\mu _{ij})$ be the random times that the first edge at *i* and the first edge *ij* appear, respectively. Thus, $N_i(t)\geqslant 1\iff T_i\leqslant t$ and $N_{ij}(t)\geqslant 1\iff T_{ij}\leqslant t$.

By the construction of $\tilde{G}_t$,

6.4 $$\begin{aligned} v(\tilde{G}_t)=v(\tilde{G}^*_t)&= \sum _i \varvec{1}_{\{N_i(t)\geqslant 1\}} =\sum _i \varvec{1}_{\{T_i\leqslant t\}},\end{aligned}$$

6.5 $$\begin{aligned} e(\tilde{G}_t)&= \sum _{i<j} \varvec{1}_{\{N_{ij}(t)\geqslant 1\}} =\sum _{i<j}\varvec{1}_{\{T_{ij}\leqslant t\}}. \end{aligned}$$

Recall that for every fixed *t*, the numbers $N_{ij}(t)$ are independent random variables, and thus the indicators in the sums (6.5) are independent. However, the numbers $N_i(t)$ and the indicators in the sums in (6.4) are dependent, which is a complication. (For example, $v(\tilde{G}_t)=1$ is impossible, since there are no isolated vertices and no loops.)

We give first a simple lemma for the type of sums in (6.5), where the terms are independent.

### Lemma 6.1

Let $Z_i\sim {\text {Exp}}(\lambda _i)$, $i=1,2,\dots$, be independent exponential random variables with $\lambda _i\geqslant 0$ and $0<\sum _{i=1}^\infty \lambda _i<\infty$, and let $W(t):=\sum _{i=1}^\infty \varvec{1}_{\{Z_i\leqslant t\}}$. Then, the following hold.

(i) For every $$t\geqslant 0$$,
  6.6 $$\begin{aligned} {\mathbb {E}}W(t) = \sum _{i=1}^\infty {\mathbb {P}}\bigl (Z_i\leqslant t\bigr ) =\sum _{i=1}^\infty \bigl (1-e^{-\lambda _i t}\bigr )<\infty \end{aligned}$$
  and thus a.s $W(t)<\infty$ for every $t\geqslant 0$. Furthermore, ${\mathbb {E}}W(t)$ is a strictly increasing and concave continuous function of $t\geqslant 0$ with ${\mathbb {E}}W(0)=0$ and ${\mathbb {E}}W(t)/t\rightarrow 0$ as ${t\rightarrow \infty }$.
(ii) For $$t>0$$,
  6.7 $$\begin{aligned} {\mathbb {E}}W(t)\asymp \sum _{i=1}^\infty \bigl (1\wedge (\lambda _i t)\bigr ). \end{aligned}$$
(iii) For every $t\geqslant 0$,
  6.8 $$\begin{aligned} {\text {Var}}\bigl (W(t)\bigr ) =\sum _{i=1}^\infty e^{-\lambda _i t} \bigl (1-e^{-\lambda _i t}\bigr ) \leqslant {\mathbb {E}}W(t). \end{aligned}$$
(iv) Let $L:=|\{i:\lambda _i>0\}|\leqslant \infty$. Then as ${t\rightarrow \infty }$, ${\mathbb {E}}W(t)\rightarrow L$, $W(t)\overset{\mathrm {a.s.}}{\longrightarrow }L$ and
  6.9 $$\begin{aligned} \frac{W(t)}{{\mathbb {E}}W(t)}\overset{\mathrm {a.s.}}{\longrightarrow }1. \end{aligned}$$
(v) If $(t_n)$ and $(t'_n)$ are two sequences of positive numbers with $t_n'/t_n\rightarrow 1$, then ${\mathbb {E}}W(t_n')/{\mathbb {E}}W(t_n)\rightarrow 1$.

### Proof

This is presumably all known, but it seems easier to give a proof than to find references. Note that *W*(*t*) is increasing as a function of *t*.

(i) The calculation (6.6) of the expectation is immediate, and the sum is finite because $1-e^{-\lambda _i t}\leqslant \lambda _i t$. Hence *W*(*t*) is a.s finite for, say, each integer *t*, and thus for all $t\geqslant 0$. It follows by (6.6) that ${\mathbb {E}}W(t)$ is strictly increasing and concave. Moreover, the sum converges uniformly on every finite interval [0, *T*], and thus ${\mathbb {E}}W(t)$ is continuous. Finally, ${\mathbb {E}}W(t)/t=\sum _{i=1}^\infty (1-e^{-\lambda _i t})/t$, where each summand tends to 0 as ${t\rightarrow \infty }$, and is bounded by $\lambda _i$. Hence ${\mathbb {E}}W(t)/t\rightarrow 0$ as ${t\rightarrow \infty }$by dominated convergence of the sum.
(ii) An immediate consequence of (6.6) and $1-e^{-x}\asymp 1\wedge x$.
(iii) Since the summands in *W* are independent,
  6.10 $$\begin{aligned} \begin{aligned} {\text {Var}}\bigl (W(t)\bigr )&=\sum _{i=1}^\infty {\mathbb {P}}(Z_i\leqslant t)\bigl (1-{\mathbb {P}}(Z_i\leqslant t)\bigr ) =\sum _{i=1}^\infty e^{-\lambda _i t} \bigl (1-e^{-\lambda _i t}\bigr ) \\&\leqslant \sum _{i=1}^\infty {\mathbb {P}}(Z_i\leqslant t)={\mathbb {E}}W(t). \end{aligned} \end{aligned}$$
(iv) First, by (6.6) and monotone convergence, as ${t\rightarrow \infty }$,
  6.11 $$\begin{aligned} {\mathbb {E}}W(t)\rightarrow \sum _{i=1}^\infty {\mathbb {P}}(Z_i<\infty )=\sum _{i=1}^\infty \varvec{1}_{\{\lambda _i>0\}}=L. \end{aligned}$$
  Furthermore, if $L<\infty$, then a.s $W(t)= L$ for all large *t*, and thus (6.9) holds. Suppose now that $L=\infty$. Then ${\mathbb {E}}W(t)\rightarrow \infty$ by (6.11). Let $\delta \in (0,1)$, let $a:=1+\delta$ and choose, for $n\geqslant 1$, $t_n>0$ such that ${\mathbb {E}}W(t_n)=a^n$. (This is possible by (i).) By (6.8) and Chebyshev’s inequality, for any $t>0$,
  6.12 $$\begin{aligned} {\mathbb {P}}\Bigl (\Bigl |\frac{W(t)}{{\mathbb {E}}W(t)}-1\Bigr |>\delta \Bigr ) \leqslant \frac{{\text {Var}}(W(t))}{(\delta {\mathbb {E}}W(t))^2} \leqslant \frac{1}{\delta ^2 {\mathbb {E}}W(t)}. \end{aligned}$$
  Hence, by our choice of $t_n$ and the Borel–Cantelli lemma, a.s there exists a (random) $n_0$ such that $1-\delta \leqslant W(t_n)/{\mathbb {E}}W(t_n)\leqslant 1+\delta$ for $n\geqslant n_0$. This, and the fact that *W*(*t*) is increasing, implies that if $t\geqslant t_{n_0}$, and we choose $n\geqslant n_0$ such that $t_n\leqslant t<t_{n+1}$, then
  6.13 $$\begin{aligned} W(t)\leqslant W(t_{n+1})\leqslant (1+\delta ) a^{n+1} =(1+\delta )^2 {\mathbb {E}}W(t_n) \leqslant (1+\delta )^2 {\mathbb {E}}W(t), \end{aligned}$$
  and similarly
  6.14 $$\begin{aligned} W(t)\geqslant W(t_{n})\geqslant (1-\delta ) a^{n} \geqslant (1-\delta )^2 {\mathbb {E}}W(t_{n+1}) \geqslant (1-\delta )^2 {\mathbb {E}}W(t). \end{aligned}$$
  Consequently, a.s
  6.15 $$\begin{aligned} (1-\delta )^2\leqslant \liminf _{{t\rightarrow \infty }} \frac{W(t)}{{\mathbb {E}}W(t)} \leqslant \limsup _{{t\rightarrow \infty }} \frac{W(t)}{{\mathbb {E}}W(t)} \leqslant (1+\delta )^2. \end{aligned}$$
  Since $\delta$ is arbitrarily small, (6.9) follows.
(v) By (i), ${\mathbb {E}}W(t)$ is increasing, and furthermore it is concave with ${\mathbb {E}}W(0)=0$, and thus ${\mathbb {E}}W(t)/t$ is decreasing on $(0,\infty )$. Hence,
  6.16 $$\begin{aligned} \min \{1,t_n'/t_n\} \leqslant {\mathbb {E}}W(t_n')/{\mathbb {E}}W(t_n) \leqslant \max \{1,t_n'/t_n\} \end{aligned}$$
  and the result follows. $\square$

In order to extend this to the dependent sum (6.4), we use a lemma.

### Lemma 6.2

Let $(I_{ij})_{i,j=1}^N$ be a finite or infinite symmetric array of random indicator variables, with $\{I_{ij}\}_{i\leqslant j}$ independent. Let $I_i:=\max _j I_{ij}$, and $W:=\sum _i I_i$. Then

6.17 $$\begin{aligned} {\text {Var}}W \leqslant 2 {\mathbb {E}}W. \end{aligned}$$

### Proof

Assume first that $N<\infty$. Let $\bar{I}_{ij}:=1- I_{ij}$ and $\bar{I}_i:=1-I_i=\prod _j\bar{I}_{ij}$. Let $q_{ij}:={\mathbb {E}}\bar{I}_{ij}=1-{\mathbb {E}}I_{ij}$.

Fix *i* and *j* with $i\ne j$, and let $\bar{I}'_i:=\prod _{k\ne j} \bar{I}_{ik}$ and $\bar{I}'_j:=\prod _{k\ne i} \bar{I}_{jk}$. Then $\bar{I}_i=\bar{I}_{ij}\bar{I}'_i$ and $\bar{I}_j=\bar{I}_{ij}\bar{I}'_j$, with $\bar{I}_{ij}$, $\bar{I}'_i$ and $\bar{I}'_j$ independent, and thus

6.18 $$\begin{aligned} \begin{aligned} {\text {Cov}}(I_i,I_j)&={\text {Cov}}(\bar{I}_i,\bar{I}_j) = {\mathbb {E}}\bigl (\bar{I}_{ij}\bar{I}'_i\bar{I}'_j\bigr ) -{\mathbb {E}}\bigl (\bar{I}_{ij}\bar{I}'_i\bigr ){\mathbb {E}}\bigl (\bar{I}_{ij}\bar{I}'_j\bigr ) \\&= q_{ij}{\mathbb {E}}\bar{I}'_i\bar{I}'_j - \bigl (q_{ij}{\mathbb {E}}\bar{I}'_i\bigr )\bigl (q_{ij}\bar{I}'_j\bigr ) = q_{ij}(1-q_{ij}){\mathbb {E}}\bar{I}'_i\bar{I}'_j . \end{aligned} \end{aligned}$$

In particular,

6.19 $$\begin{aligned} {\text {Cov}}(I_i,I_j)\leqslant (1-q_{ij}){\mathbb {E}}\bar{I}'_i ={\mathbb {P}}\bigl (I_{ij}=1, \, I_{ik}=0\, \text { for }\, k\ne j\bigr ). \end{aligned}$$

Summing over *j*, we obtain for every *i*, since the events $\mathcal E_j:=\{I_{ij}=1, \, I_{ik}=0 \,\text { for }\, k\ne j\}$ in (6.19) are disjoint and with union $\{\sum _jI_{ij}=1\}=\{I_i=1\}$,

$$\begin{aligned} \begin{aligned} \sum _{j\ne i}{\text {Cov}}(I_i,I_j) \leqslant \sum _{j\ne i}(1-q_{ij}){\mathbb {E}}\bar{I}'_i ={\mathbb {P}}\Bigl (\sum _j I_{ij}=1\Bigr ) \leqslant {\mathbb {P}}(I_i=1) ={\mathbb {E}}I_i. \end{aligned} \end{aligned}$$

Furthermore, ${\text {Cov}}(I_i,I_i)={\mathbb {E}}I_i - ({\mathbb {E}}I_i)^2\leqslant {\mathbb {E}}I_i$. Consequently, for every *i*,

6.20 $$\begin{aligned} \sum _{j=1}^n{\text {Cov}}(I_i,I_j) \leqslant 2 {\mathbb {E}}I_i, \end{aligned}$$

and (6.17) follows by summing over *i*.$\square$

### Lemma 6.3

Let $(Z_{ij})_{ij}$ be a symmetric array of exponential random variables with $\{Z_{ij}\}_{i\leqslant j}$ independent and $Z_{ij}\sim {\text {Exp}}(\lambda _{ij})$, where $\lambda _{ij}\geqslant 0$ and $0<\sum _{ij}\lambda _{ij}<\infty$. Let $Z_i:=\inf _j Z_{ij}$ and $W(t):=\sum _i \varvec{1}_{\{Z_i\leqslant t\}}$. Then $Z_i\sim {\text {Exp}}(\lambda _i)$ with $\lambda _i:=\sum _j\lambda _{ij}$. Moreover, all results of Lemma 6.1 hold except (iii), which is replaced by

6.21 $$\begin{aligned} {\text {Var}}\bigl (W(t)\bigr )\leqslant 2{\mathbb {E}}W(t). \end{aligned}$$

### Proof

It is well-known and elementary that $Z_i\sim {\text {Exp}}(\lambda _i)$, since $(Z_{ij})_j$ are independent for every *i*. Parts (i), (ii) and (v) of Lemma 6.1 deal only with the expectation, and their proofs do not need $Z_i$ to be independent.

Lemma 6.2 yields (6.21).

Finally, the proof of (iv) holds as before, now using (6.21). $\square$

We return to the random graphs. We define, for a given measure (matrix) $\mu$, using Lemmas 6.1 and 6.3 together with (6.2)–(6.5), the functions

6.22 $$\begin{aligned} v(t)&=v(t;\mu ):={\mathbb {E}}v(\tilde{G}_t) =\sum _{i=1}^\infty \bigl (1-e^{-\mu _i t}\bigr )\asymp \sum _{i=1}^\infty \bigl (1\wedge (\mu _it)\bigr ), \end{aligned}$$

6.23 $$\begin{aligned} e(t)&=e(t;\mu ):={\mathbb {E}}e(\tilde{G}_t)=\sum _{i<j}\bigl (1-e^{-\mu _{ij}t}\bigr ) \asymp \sum _{i\ne j}\bigl (1\wedge (\mu _{ij}t)\bigr ). \end{aligned}$$

Since $\tilde{G}_t$ has no isolated vertices, $e(\tilde{G}_t)\geqslant \frac{1}{2}v(\tilde{G}_t)$, and thus, cf. (6.1),

6.24 $$\begin{aligned} e(t)\geqslant \tfrac{1}{2} v(t). \end{aligned}$$

### Theorem 6.4

Assume that $\mu =(\mu _{ij})_{i,j=1}^\infty$ is a symmetric non-negative matrix with $\mu _{ii}=0$ and $0<\Vert \mu \Vert :=\sum _{i<j}\mu _{ij}<\infty$.

(i) As $${t\rightarrow \infty }$$,
  6.25 $$\begin{aligned} v(\tilde{G}_t)/v(t)&\overset{\mathrm {a.s.}}{\longrightarrow }1,\end{aligned}$$
  6.26 $$\begin{aligned} e(\tilde{G}_t)/e(t)&\overset{\mathrm {a.s.}}{\longrightarrow }1. \end{aligned}$$
  Moreover, if $\mu _{ij}>0$ for infinitely many pairs (*i*, *j*), then as ${t\rightarrow \infty }$, $v(t)\rightarrow \infty$, $e(t)\rightarrow \infty$ and $v(\tilde{G}_t),e(\tilde{G}_t)\overset{\mathrm {a.s.}}{\longrightarrow }\infty$.
(ii) As $${m\rightarrow \infty }$$,
  6.27 $$\begin{aligned} v(G_m)/v\bigl (\Vert \mu \Vert ^{-1}m\bigr )&\overset{\mathrm {a.s.}}{\longrightarrow }1,\end{aligned}$$
  6.28 $$\begin{aligned} e(G_m)/e\bigl (\Vert \mu \Vert ^{-1}m\bigr )&\overset{\mathrm {a.s.}}{\longrightarrow }1. \end{aligned}$$
  In particular, a.s.
  6.29 $$\begin{aligned} v(G_m)&\asymp \sum _{i=1}^\infty \bigl (1\wedge (\mu _im)\bigr ), \end{aligned}$$
  6.30 $$\begin{aligned} e(G_m)&\asymp \sum _{i,j}\bigl (1\wedge (\mu _{ij}m)\bigr ). \end{aligned}$$
  Consequently, if $\mu _{ij}>0$ for infinitely many pairs (*i*, *j*), then as ${m\rightarrow \infty }$, a.s $v(G_m),e(G_m)\rightarrow \infty$.

### Proof

(i) This is an immediate consequence of Lemma 6.1 (iv) and Lemma 6.3.
(ii) Part (i) and Propositions 4.9 and 4.13 show that $v(G_m)/v(\tau _m)=v(\tilde{G}_{\tau _m})/v(\tau _m)\overset{\mathrm {a.s.}}{\longrightarrow }1$. Furthermore, $\tau _m\sim \Vert \mu \Vert ^{-1}m$ by (4.9), and thus $v(\tau _m)\sim v\bigl (\Vert \mu \Vert ^{-1}m\bigr )$ by Lemmas 6.1 (v) and 6.3. Hence (6.27) follows. The proof of (6.28) is the same. Finally (6.29)–(6.30) follow by (6.22)–(6.23), and the final sentence follows by monotone convergence (or by Lemma 6.1 (iv)). $\square$

Hence, to find asymptotics of the numbers of vertices and edges in our random graphs, it suffices to study the expectations in (6.22)–(6.23). In particular, we note the following consequences.

### Corollary 6.5

Assume that $\mu =(\mu _{ij})_{i,j=1}^\infty$ is a symmetric non-negative matrix with $\mu _{ii}=0$ and $0<\Vert \mu \Vert :=\sum _{i<j}\mu _{ij}<\infty$. Then:

(i) $G_m$ is a.s dense if and only if $e(t)\asymp v(t)^2$ as ${t\rightarrow \infty }$.
(ii) $G_m$ is a.s sparse if and only if $e(t)=o(v(t)^2)$ as ${t\rightarrow \infty }$.
(iii) $G_m$ is a.s extremely sparse if and only if $e(t)\asymp v(t)$ as ${t\rightarrow \infty }$.

### Proof

By Theorem 6.4(ii). $\square$

### Corollary 6.6

Assume that $\mu =(\mu _{ij})_{i,j=1}^\infty$ is a symmetric non-negative matrix with $\mu _{ii}=0$ and $0<\Vert \mu \Vert :=\sum _{i<j}\mu _{ij}<\infty$. Then, a.s ,

(i) $$v(G_m)=o(m)$$ and $$e(G_m)=o(m)$$ as $${m\rightarrow \infty }$$;
(ii) $v(\tilde{G}_t)=o(t)$ and $e(\tilde{G}_t)=o(t)$ as ${t\rightarrow \infty }$.

### Proof

By Theorem 6.4, since $e(t)/t\rightarrow 0$ and $v(t)/t\rightarrow 0$ as ${t\rightarrow \infty }$  by Lemma 6.1 (i) and Lemma 6.3. $\square$

### Remark 6.7

If we consider the random multigraph $G^*_m$ we have (at least in the loop-less case, and in general with a minor modification) $v(G^*_m)=v(G_m)=o(m)$ by Corollary 6.6, while by definition there are *m* edges. Hence, the average degree $2e(G^*_m)/v(G^*_m)\rightarrow \infty$ a.s as ${m\rightarrow \infty }$. Similarly, the average number of copies of each edge $e(G^*_m)/e(G_m)\rightarrow \infty$ a.s

For future use we note also that since *v*(*t*) is concave with $v(0)=0$, for any $C\geqslant 1$,

6.31 $$\begin{aligned} v(t)\leqslant v(Ct)\leqslant Cv(t). \end{aligned}$$

Hence $v(Ct)\asymp v(t)$ for any constant $C>0$.

We have so far considered only simple first order properties of $v(G_m)$ and $e(G_m)$. For the number of edges, much more follows from the central limit results in the references mentioned above. In particular, the local and global central limit theorems in [19] apply and yield the following. (Although the estimates (6.32) and (6.35) are uniform in all *x*, the main interest is for *x* constant, or perhaps tending to infinity very slowly.)

### Theorem 6.8

Let $\mu$ be as in Theorem 6.4. The following hold with *O*(1) bounded by an absolute constant *C* uniformly for all $m\geqslant 1$, $x\in \mathbb R$, and matrices $\mu$.

Let $$\sigma ^2_m:={\text {Var}}(e(G_m))$$. Then

6.32 $$\begin{aligned} {\mathbb {P}}\bigl (e(G_m)=\lfloor {\mathbb {E}}e(G_m)+x\sigma _m\rfloor \bigr ) =\frac{e^{-x^2/2}}{\sqrt{2\pi }\sigma _m} + \frac{O(1)}{\sigma ^2_m}. \end{aligned}$$

Moreover, assuming for simplicity $\Vert \mu \Vert =1$,

6.33 $$\begin{aligned} {\mathbb {E}}e(G_m)&={\mathbb {E}}e(\tilde{G}_m)+O(1) ,\end{aligned}$$

6.34 $$\begin{aligned} {\text {Var}}(e(G_m))&={\text {Var}}( e(\tilde{G}_m))+O(1) , \end{aligned}$$

and, recalling (6.23) and defining $\tilde{\sigma }^2_t:={\text {Var}}(e(\tilde{G}_t))$,

6.35 $$\begin{aligned} {\mathbb {P}}\bigl (e(G_m)=\lfloor e(m)+x\tilde{\sigma }_m\rfloor \bigr ) =\frac{e^{-x^2/2}}{\sqrt{2\pi }\tilde{\sigma }_m} + \frac{O(1)}{\tilde{\sigma }^2_m}. \end{aligned}$$

In particular, if ${m\rightarrow \infty }$ and $\tilde{\sigma }^2_m\rightarrow \infty$, then $(e(G_m)-{\mathbb {E}}e(G_m))/\sigma _m\overset{\mathrm {d}}{\longrightarrow }N(0,1)$ and $(e(G_m)-e(m))/\tilde{\sigma }_m\overset{\mathrm {d}}{\longrightarrow }N(0,1)$.

The *O*(1) in (6.33)–(6.34) can be replaced by *o*(1) as ${m\rightarrow \infty }$ for a fixed $\mu$.

### Proof

By [19, Theorems 2.1, 2.3, 2.4 and Corollary 2.5, together with Sect. 9]. $\square$

Note that $e(m)={\mathbb {E}}e(\tilde{G}_m)$ and $\tilde{\sigma }^2_m={\text {Var}}e(\tilde{G}_m)$ are given by (6.23) and (6.8); they are usually simpler and more convenient to handle than ${\mathbb {E}}e(G_m)$ and $\sigma ^2_m={\text {Var}}(e(G_m))$.

We conjecture that similar results holds for $v(G_m)$, the number of vertices. However, we cannot obtain this directly from results on the occupancy problem in the same way as Theorem 6.8, again because the variables $N_i(t)$ are dependent. (The number of vertices corresponds to an occupancy problem where balls are thrown in pairs, with a dependency inside each pair.)

### Problem 6.9

Show asymptotic normality for $v(G_m)$ when ${\text {Var}}(v(G_m))\rightarrow \infty$.

### The Case with Dust or Attached Stars

We consider briefly the case when the model contains dust (other than loops) or attached stars. In this case, the results are quite different. We may for simplicity assume that there are no loops at all, since loops are deleted in any case. Thus $\mu _{ii}=0$ for $i\geqslant 0$ and $\mu _{0i}>0$ for some $i\in \mathbb N\cup \{-1\}$.

The number of edges in the dust and attached stars of $\tilde{G}_t$ is ${\text {Po}}(ct)$ with $c:=\sum _{i=-1}^\infty \mu _{0i}>0$, and thus this number is a.s $\sim ct\asymp t$ as ${t\rightarrow \infty }$, by the law of large numbers for the Poisson process. (Recall that all edges in the dust and attached stars of $\tilde{G}^*_t$ are simple, so the number of them is the same in $\tilde{G}_t$ and in $\tilde{G}^*_t$.) It follows by Proposition 4.9 that the number of edges in the dust and attached stars of $G_m$ a.s is $\asymp m$. Moreover, since each edge in the dust or an attached star has at least one endpoint that is not shared by any other edge, the same estimates hold for the number of vertices in the dust and attached stars. This leads to the following theorem, which shows that if there is any dust or attached star all, then those parts will dominate the random graphs.

#### Theorem 6.10

Assume that $\mu _{0i}>0$ for some $i\in \mathbb N\cup \{-1\}$. Then, a.s ,

(i) $$v(G_m)\asymp m$$ and $$e(G_m)\asymp m$$ as $${m\rightarrow \infty }$$;
(ii) $v(\tilde{G}_t)\asymp t$ and $e(\tilde{G}_t)\asymp t$ as ${t\rightarrow \infty }$.

Moreover, a.s , all but a fraction *o*(1) of the edges and vertices are in the dust or attached stars.

Consequently, the random graphs $G_m$ are a.s extremely sparse, but in a rather trivial way.

#### Proof

The argument before the theorem shows (i) and (ii).

Moreover, Corollary 6.6 applies to the central part of $\tilde{G}_t$ and shows that the number of edges and vertices there a.s are *o*(*t*), and thus only a fraction *o*(1) of all edges and vertices. By Proposition 4.9, the same holds for $G_m$. $\square$

## Rank 1 Multigraphs

We turn to considering specific examples of the construction. One interesting class of examples are constructed as follows.

### Example 7.1

(Rank 1) Let $(q_i)_1^\infty$ be a probability distribution on $\mathbb N$, and construct a sequence of i.i.d. edges $e_1,e_2,\dots$, each obtained by selecting the two endpoints as independent random vertices with the distribution $(q_i)_i$. (Thus loops are possible.) Define the random multigraph $G^*_m$ by taking the *m* edges $e_1,\dots ,e_m$, letting the vertex set be the set of their endpoints. (Equivalently: start with the vertex set $\mathbb N$ and then remove all isolated vertices.)

In other words, let $V_1,V_2,\dots$ be an i.i.d. sequence of vertices with the distribution $(q_i)_i$, and let the edges of $G^*_m$ be $V_1V_2, V_3V_4, \dots ,V_{2m-1}V_{2m}$.

This is clearly a random multigraph of the type constructed in Sect. 5, with

7.1 $$\begin{aligned} \mu _{ij}= {\left\{ \begin{array}{ll} 2q_iq_j,&{}i\ne j, \\ q_i^2, &{}i=j. \end{array}\right. } \end{aligned}$$

We thus have, by (5.2),

7.2 $$\begin{aligned} \mu _i=\sum _{j\ne i} 2q_iq_j+q_i^2=2q_i-q_i^2. \end{aligned}$$

In particular, $$\mu _i\asymp q_i$$.

The corresponding Poisson model $\tilde{G}^*_t$ is by Proposition 4.9 obtained by taking a Poisson number of edges $e_1,\dots ,e_{N(t)}$, with $N(t)\sim {\text {Po}}(t)$.

As usual, we obtain the corresponding simple graphs by omitting all repeated edges and deleting all loops.

We call a random multigraph constructed as in Example 7.1, or equivalently by (7.1), for some (possibly random) probability distribution $(q_i)_1^\infty$, a *rank 1 edge exchangeable multigraph*, for the reason that the matrix (7.1) is a rank 1 matrix except for the diagonal entries.

### Remark 7.2

The diagonal entries, creating loops, are less important to us. In the multigraph examples below, it is natural, and simplifies the results, to allow loops. However, when we consider the simple graphs $\tilde{G}_t$ and $G_m$, we ignore loops and, see Remark 5.1, it is then simpler to modify (7.1) by taking $\mu _{ii}=0$; we still say that the resulting random graphs are rank 1.

### Remark 7.3

Note that the rank 1 random graphs in [2] are different; they are simple graphs, and they are vertex exchangeable or modifications of vertex exchangeable random graphs, cf. Sect. 5.1. Nevertheless, both types of “rank 1” random graphs can be seen as based on the same idea: each vertex is given an “activity” ($q_i$ in our case), and the probability of an edge between two vertices is proportional to the product of their activities. (See the references in [2] for various versions of this idea.)

Recall that the *configuration model* is an important model for constructing random multigraphs with a given degree sequence, which is defined as follows, see e.g. [1].

### Definition 7.4

(*Configuration model*) Given a sequence $(d_i)_{i=1}^n$ of non-negative integers with $\sum _id_i$ even, the random multigraph $\tilde{G}^*(n,(d_i)_{i=1}^n)$is defined by considering a set of $\sum _i d_i$ *half-edges* (or *stubs*), of which $d_i$ are labelled *i* for each $i\in [n]$, and taking a uniformly random matching of the half-edges; each pair of half-edges is interpreted as an edge between the corresponding vertices.

By construction, the multigraph $\tilde{G}^*(n,(d_i)_{i=1}^n)$has degree sequence $(d_i)_{i=1}^n$. (With a loop counted as 2 edges at its only endpoint.) Note that the distribution of $\tilde{G}^*(n,(d_i)_{i=1}^n)$ is not uniform over all multigraphs with this degree sequence. (As is well-known, and easy to see, the probability distribution has a factor (weight) 1 / 2 for each loop and $1/\ell !$ for each edge of multiplicity $\ell >1$; in particular, conditioned on being a simple graph, $\tilde{G}^*(n,(d_i)_{i=1}^n)$ has a uniform distribution.) Nevertheless, $\tilde{G}^*(n,(d_i)_{i=1}^n)$ has the right distribution for our purposes.

### Theorem 7.5

The random multigraph $G^*_m$ constructed in Example 7.1 has, conditioned on its degree sequence $(d_i)_{i=1}^n$, the same distribution as the random multigraph $\tilde{G}^*(n,(d_i)_{i=1}^n)$ constructed by the configuration model for that degree sequence.

The same holds for $\tilde{G}^*_t$.

### Proof

In the construction of $G^*_m$ above, the sequence $V_1,\dots ,V_{2m}$ is i.i.d., and thus exchangeable; hence its distribution is unchanged if we replace each $V_i$ by $V_{\pi (i)}$ for a uniformly random permutation $\pi$ of [2*m*], independent of everything else. Consequently, the distribution of $G^*_m$ is the same if we modify the definition above and let the edges be $V_{\pi (1)}V_{\pi (2)},\dots ,V_{\pi (2m-1)}V_{\pi (2m)}$; but this is the same as saying that the edges are obtained by taking a random matching of the multiset $\{V_1,\dots ,V_{2m}\}$, which is precisely what the configuration model does. (Note that the vertex degree $d_i$ is the number of times *i* appears in $V_1,\dots ,V_{2m}$.)

The result for $\tilde{G}^*_t$ follows, since the degree sequence tells how many edges there are, so conditioning on the degree sequence implies conditioning on $e(\tilde{G}^*_t)=N(t)$, which reduces to the case of $G^*_m$ just proved, see Remark 4.10. $\square$

### Remark 7.6

In statistical language, the theorem implies that the degree distribution is a sufficient statistic for the family of distributions of multigraphs $G^*_m$ (or $\tilde{G}^*_t$) given by Example 7.1 with different distributions $(q_i)_1^\infty$.

### Example 7.7

A trivial example of the construction in Example 7.1 is obtained by fixing $n\geqslant 1$ and letting $q_i=1/n$, $1\leqslant i\leqslant n$, i.e., the uniform distribution on [*n*]. This means that we consider a sequence of i.i.d. edges, each obtained by taking the two endpoints uniformly at random, and independently, from [*n*]. In other words, the endpoints of the edges are obtained by drawing with replacement from [*n*]. This gives the random multigraph process studied in e.g. [25], which is a natural multigraph version of the (simple) random graph process studied by [16].

The rank 1 random multigraphs in Example 7.1 appear also hidden in some other examples.

### Example 7.8

(The Hollywood model) The Hollywood model of a random hypergraph was defined in [11] using the language of actors participating in the same movie, see [11] for details. We repeat their definition in somewhat different words.

The model can be defined by starting with the two-parameter version of the Chinese restaurant process, see e.g. [35, Sect. 3.2] and [10], which starts with a single table with one customer. New customers arrive, one by one; if a new customer arrives when there are *n* customers seated at *k* tables, with $n_i\geqslant 1$ customers at table *i*, then the new customer is placed:

7.3 $$\begin{aligned} \left\{ \begin{array}{llll} &{}\text {at table}\, i (1\leqslant i\leqslant k)\, \text {with probability}\quad &{}&{} (n_i-\alpha )/(n+\theta ), \\ &{}\text {at a new table}\, k+1\, \text {with probability}&{}&{} (\theta +k\alpha )/(n+\theta ). \end{array} \right. \end{aligned}$$

Here $\alpha$ and $\theta$ are parameters, and either

(i) $$0\leqslant \alpha \leqslant 1$$ and $$\theta >-\alpha $$, or
(ii) $\alpha <0$ and $\theta =N|\alpha |>0$ for some $N\in \mathbb N$.

In case (ii), there are never more than *N* tables; in case (i), the number of tables grows a.s to $\infty$.

In the construction of the Hollywood model hypergraph, the vertices are the tables in the Chinese restaurant process. We furthermore draw the sizes of the edges as i.i.d. random variables $X_j$ with some distribution $\nu$ on the non-negative integers $\mathbb N$. The first edge is then defined by (the set of tables of) the first $X_1$ customers, the second edge by the next $X_2$ customers, and so on. The random hypergraph $\tilde{G}_m$ with *m* edges is thus described by the first $X_1+\dots +X_m$ customers.

A standard calculation shows that the sequence of table numbers is exchangeable, except that the numbers occur for the first time in the natural order; to be precise, the probability of any finite sequence of table numbers, such that the first 1 appears before the first 2, and so on, depends only on the number of occurences of each number. Consequently, as noted in [11], since we ignore vertex labels, and the sequence $X_1,X_2,\dots$ is i.i.d. and independent of the Chinese restaurant process, the random hypergraph $\tilde{G}^*_\infty$ is exchangeable, and by the representation theorem by [11, 12], see Remark 4.4, the Hollywood model can be constructed as in Definition 4.2 for some random measure $\mu$ on $\mathbb N$.

We can see this more concretely by replacing the table labels $i\in \mathbb N$ by i.i.d. random labels $U_i\sim U(0,1)$; then the sequence of table labels of the customers is exchangeable. Hence, by de Finetti’s theorem, there exists a random probability measure $\hat{P}$ on $[0,1]$ such that conditioned on $\hat{P}$, the sequence of (new) table labels is an i.i.d. sequence with distribution $\hat{P}$. Clearly, the random measure $\hat{P}=\sum _i \widetilde{P}_i\delta _{U_i}$ for some random sequence $\widetilde{P}_i$ of numbers with $\sum _i\widetilde{P}_i=1$. Furthermore, by the law of large numbers, for every $i\in \mathbb N$, $\widetilde{P}_i$ equals a.s the asymptotic frequency of customers sitting at the table originally labelled *i* in the Chinese restaurant process. Hence, the random probability measure $\widetilde{P}=(\widetilde{P}_i)_1^\infty$ on $\mathbb N$ has the distribution $\mathrm {GEM}(\alpha ,\theta )$, see [35, Theorem 3.2 and Definition 3.3]. (An alternative version of this argument uses Kingman’s paintbox representation for exchangeable random partitions [35, Theorem 2.2] instead of the random lables $U_i$ above; we leave the details to the interested reader.) Consequently, the Hollywood model hypergraph can be constructed as follows: Let the random probability measure $\widetilde{P}$ on $\mathbb N$ have the distribution $\mathrm {GEM}(\alpha ,\theta )$; conditionally given $\widetilde{P}$ take an infinite i.i.d. sequence of vertices with distribution $\widetilde{P}$; construct the edges by taking the first $X_1$ vertices, the next $X_2$ vertices, ...; finally, ignore the vertex labels.

We specialize to the graph case and assume from now on that $X_j=2$ (deterministically). Thus edges are constructed by taking the customers pairwise as they arrive. We then see by comparing the constructions above and in Example 7.1 that the Hollywood model yields the same result as the rank 1 model in Example 7.1, based on a random probability distribution with distribution $\mathrm {GEM}(\alpha ,\theta )$.

Since the order of the probabilities $q_i$ does not matter in Example 7.1, we obtain the same result if we reorder the probabilities $\widetilde{P}_i$ in decreasing order; this gives the Poisson–Dirichlet distribution $\mathrm {PD}(\alpha ,\theta )$ [35, Definition 3.3], and thus the Hollywood model is also given by the rank 1 model based on $\mathrm {PD}(\alpha ,\theta )$.

Theorem 7.5 shows that yet another way to define the Hollywood model multigraph $G^*_m$ is to take the configuration model where the degree sequence $(d_i)_1^m$ is the (random) sequence of numbers of customers at each table in the Chinese restaurant process when there are 2*m* customers.

### Example 7.9

[36] considers the random multigraph process with a fixed vertex set [*N*], where edges are added one by one (starting with no edges) such that the probability that a new edge joins two distinct vertices *i* and *j* is proportional to $2(d_i+\alpha )(d_j+\alpha )$, and the probabiity that the new edge is a loop at *i* is proportional to $(d_i+\alpha )(d_i+1+\alpha )$; here $d_i$ is the current degree of vertex *i* and $\alpha >0$ is a fixed parameter. ([36] considers also the corresponding process for simple graphs; we do not consider that process here.)

It is easily seen that this multigraph process can be obtained as above, with a minor modification of the Chinese restaurant process. Consider now a restaurant with a fixed number *N* of tables, initially empty, and seat each new customer at table *i* with probability

7.4 $$\begin{aligned} (n_i+\alpha )/(n+N\alpha ), \end{aligned}$$

where $n_i\geqslant 0$ is the number of customers at table *i* and *n* is their total number. Then construct edges by taking the customers pairwise, as above; this yields the multigraph process just described.

Furthermore, although this construction uses a modification of the Chinese restaurant process, we can relabel the tables in the random order that they are occupied. It is then easily seen that we obtain the Chinese restaurant process (7.3) with parameters $(-\alpha ,N\alpha )$. Since the vertex labels are ignored, this means that Pittel’s multigraph process is the same as the Hollywood model with parameters $(-\alpha ,N\alpha )$. Consequently, it can be defined by the rank 1 model in Example 7.1 with the random probability distribution $\mathrm {GEM}(-\alpha ,N\alpha )$ on $[N]\subset \mathbb N$, or, equivalently, the random probability distribution $\mathrm {PD}(-\alpha ,N\alpha )$.

Moreover, the restaurant process (7.4) can be seen as a Pólya urn process, with balls of *N* different colours and initially $\alpha$ balls in each colour, where $n_i$ is the number of additional balls of color *i* in the urn; balls are drawn uniformly at random from the urn, and each drawn ball is replaced together with a new ball of the same colour. Note that then $n_i$ is the number of times colour *i* has been drawn. (It does not matter whether $\alpha$ is an integer or not; the extension to non-integer $\alpha$ causes no mathematical problem, see e.g. [20, Remark 4.2], [21] or [28].) The sequence of vertex labels is thus given by the sequence of colours of the balls drawn from this urn. It is well-known, by an explicit calculation, see e.g. [33] (where $N=2$), that this sequence is exchangeable. By de Finetti’s theorem it can thus can be seen as an i.i.d. sequence of colours with a random distribution $\hat{P}$, which equals the asymptotic colour distribution. Moreover, it is well-known [29] (see also [33, 37] for $N=2$) that this asymptotic distribution is a symmetric Dirichlet distribution ${\text {Dir}}(\alpha /N,\dots ,\alpha /N)$, with the density function $c\prod x_i^{\alpha /N-1}$ on the $(N-1)$-dimensional simplex $\{(x_1,\dots ,x_N)\in \mathbb R_+^N:\sum _i x_i=1$}. Consequently, the multigraph process $\tilde{G}^*_N$ can be obtained by the rank 1 model in Example 7.1 with the random probability distribution ${\text {Dir}}(\alpha /N,\dots ,\alpha /N)$.

Alternatively, by Theorem 7.5, $G^*_m$ may be obtained by the configuration model, with vertex degrees given by the first 2*m* draws in the Pólya urn process described above.

See further [27].

### Rank 1 Simple Graphs

We will in the following sections study several examples of the simple random graphs $G_m$ in the rank 1 case. We note here a few general formulas. We ignore the trivial case when the probability distribution $\{q_i\}$ is supported on one point. (Then $\tilde{G}_t$ and $G_m$ have only a single vertex and no edges. In fact, the interesting case is when the support of $\{q_i\}$ is infinite.) We thus assume $\max q_i<1$.

Since we ignore loops when constructing the simple graphs $\tilde{G}_t$ and $G_m$, we modify (7.1) by taking $\mu _{ii}=0$, see Remark 7.2; this changes (7.2) to $\mu _i=2q_i-2q_i^2$, but we still have $\mu _i\asymp q_i$. Thus (6.22) and (6.23) yield

7.5 $$\begin{aligned} v(t)&\asymp \sum _i \bigl (1\wedge (tq_i)\bigr ),\end{aligned}$$

7.6 $$\begin{aligned} e(t)&\asymp \sum _{i\ne j} \bigl (1\wedge (tq_iq_j)\bigr ) . \end{aligned}$$

Moreover, adding the diagonal terms to the sum in (7.5) does not affect this estimate, since if we assume as we may that $q_1,q_2>0$, then $q_i^2=O(q_1q_i)$ and $q_1^2=O(q_1q_2)$, and thus $\sum _{i} \bigl (1\wedge (tq_i^2)\bigr ) =O\bigl (\sum _{i>1} \bigl (1\wedge (tq_1q_i)\bigr )\bigr ) =O\bigl (e(t)\bigr )$. Hence also

7.7 $$\begin{aligned} e(t)&\asymp \sum _{i, j} \bigl (1\wedge (tq_iq_j)\bigr ) \asymp \sum _i v(tq_i) . \end{aligned}$$

Note that although we are interested in large *t*, the argument $tq_i$ in (7.7) is small for large *i*, so (7.7) requires that we consider *v*(*t*) for both large and small *t*.

Similarly, the expected degree of vertex *i* in $\tilde{G}_t$ is

7.8 $$\begin{aligned} \begin{aligned} {\mathbb {E}}D_i&= \sum _{j\ne i} \bigl (1-e^{-2tq_iq_j}\bigr ) \asymp \sum _{j\ne i} \bigl (1\wedge (tq_iq_j)\bigr ) \asymp \sum _{j} \bigl (1\wedge (tq_iq_j)\bigr ) \asymp v(tq_i). \end{aligned} \end{aligned}$$

## Dense Examples

We may obtain examples where $G_m$ and $\tilde{G}_t$ are dense by letting $\mu _{ij}$ decrease very rapidly.

We begin with an extreme case, which gives complete graphs.

### Example 8.1

(Complete graphs) Let $\mu =(\mu _{ij})$ be such that for every $k\geqslant 2$

8.1 $$\begin{aligned} 0< \sup _{\ell \geqslant 1}\mu _{k+1,\ell } \leqslant k^{-4}\min _{i<k}\mu _{k,i}. \end{aligned}$$

For example, we may take $\mu _{ij}=((i\vee j)!)^{-4}$, or the rank 1 example $\mu _{ij}=q_iq_j$ with $q_i=\exp (-3^i)$.

We will show that a.s , for all large *n*, $$G_{\left( {\begin{array}{c}n\\ 2\end{array}}\right) }$$ is the complete graphs $K_n$.

Define $a_i:=\sup _{j}\mu _{ij}$. Then (8.1) implies, for every $k\geqslant 2$,

8.2 $$\begin{aligned} a_{k+1}\leqslant k^{-4}\mu _{k1}\leqslant k^{-4} a_k. \end{aligned}$$

In particular, for $k\geqslant 2$, $a_{k+1}\leqslant \frac{1}{2}a_k$. Moreover, $(k-1)^4a_k \geqslant a_{k}\geqslant k^4a_{k+1}$; hence the sequence $k^4a_{k+1}$ is decreasing for $k\geqslant 1$.

Define $t_n:=(n^3 a_{n+1})^{-1}$. Let $Y_n$ be the number of edges in $\tilde{G}^*_{t_n}$ with at least one endpoint outside [*n*]. Then, since (8.2) implies that $(k+1)a_{k+1}\leqslant \frac{1}{2}ka_k$ when $k\geqslant 2$,

8.3 $$\begin{aligned} {\mathbb {E}}Y_n =\sum _{k\geqslant n+1}\sum _{i<k} t_n\mu _{ki} <t_n\sum _{k\geqslant n+1}k a_k \leqslant 2t_n (n+1) a_{n+1} =\frac{2(n+1)}{n^3}. \end{aligned}$$

Consequently, by Markov’s inequality and the Borel–Cantelli lemma, a.s $Y_n=0$ for all large *n*.

On the other hand, if $Z_n$ is the number of pairs (*i*, *j*) with $i<j\leqslant n$ such that *ij* is not an edge of $\tilde{G}^*_{t_n}$, i.e., $N_{ij}(t_n)=0$, then

8.4 $$\begin{aligned} {\mathbb {E}}Z_n = \sum _{i<j\leqslant n} {\mathbb {P}}(N_{ij}(t_n)=0) = \sum _{i<j\leqslant n} e^{-t_n\mu _{ij}}. \end{aligned}$$

Moreover, if $i<j\leqslant n$, then by (8.1) and (8.2), $\mu _{ij}\geqslant j^4 a_{j+1}\geqslant n^4a_{n+1}$ and thus $t_n\mu _{ij}\geqslant t_n n^4a_{n+1}=n$. Hence, (8.4) yields $${\mathbb {E}}Z_n \leqslant \left( {\begin{array}{c}n\\ 2\end{array}}\right) e^{-n}$$, and we see, by the Borel–Cantelli lemma again, that a.s also $Z_n=0$ for all large *n*.

We have shown that a.s for all large *n*, $\tilde{G}^*_{t_n}$ contains at least one edge *ij* whenever $i<j\leqslant n$, but no other edges; in other words, the simple graph $\tilde{G}_{t_n}$ is the complete graph $K_n$. Since $K_n$ has $$\left( {\begin{array}{c}n\\ 2\end{array}}\right) $$ edges, this also means that $$G_{\left( {\begin{array}{c}n\\ 2\end{array}}\right) }=K_n$$, as asserted above.

We have shown that a.s , for all large *m*, $G_m$ is the complete graph $K_n$ if $$m=\left( {\begin{array}{c}n\\ 2\end{array}}\right) $$; since $G_n$ is an increasing sequence of graphs, it follows that for intermediate values $$m=\left( {\begin{array}{c}n\\ 2\end{array}}\right) +\ell $$, $1\leqslant \ell <n$, $G_m$ consist of $K_n$ plus an additional vertex joined to $\ell$ of the other vertices. We thus have a complete description of the process $(G_m)$ for large *m*. (And thus also of the process $\tilde{G}_t$.)

In particular, for all large *m*, $G_m$ differs from the complete graph $K_n$ with $n=v(G_m)$ by less than *n* edges, and thus, see Sect. 3, $\delta _{\square }(G_m,K_n)\leqslant \Vert W_{G_m}-W_{K_n}\Vert _{L^1}\leqslant 2/n=o(1)$. It follows that in the sense of graph limit theory, $G_m\rightarrow \Gamma _1$ a.s , where $\Gamma _1$ is the graph limit defined as the limit of the complete graphs, which is the graph limit defined by the constant graphon $W_1(x,y)=1$ (on any probability space $\Omega$).

The assumption (8.1) in Example 8.1 is is not best possible, and may easily be improved somewhat, but we only wanted to give a class of examples.

### Problem 8.2

Find necessary and sufficient conditions on $\mu$ for $G_m$ to be complete for all large *m* of the form $$m=\left( {\begin{array}{c}n\\ 2\end{array}}\right) $$.

Here is another example, where the limit is less trivial.

### Example 8.3

Consider a rank 1 example $\mu _{ij}=q_iq_j$, $i\ne j$, where $q_i$ has a geometric decay $q_i\asymp b^{-i}$ for some $b>1$.

Let $n\geqslant 1$ and suppose $b^n\leqslant t\leqslant b^{n+1}$. Then the expected number of edges *ij* in $\tilde{G}_t$ with $i+j>n$ is at most, with $C:=\sup _i b^iq_i<\infty$ and letting $\ell =i+j$,

8.5 $$\begin{aligned} \sum _{i+j>n}t q_iq_j \leqslant t\sum _{i+j>n} C^2 b^{-i}b^{-j} \leqslant b^{n+1}\sum _{\ell \geqslant n+1}\ell C^2 b^{-l} =O(n). \end{aligned}$$

Similarly, the expected number of edges *ij* with $i+j\leqslant n$ *not* in $\tilde{G}_t$ is at most, for $c:=\inf _i b^iq_i>0$,

8.6 $$\begin{aligned} \begin{aligned} \sum _{i+j\leqslant n}\exp (-2t q_iq_j)&\leqslant \sum _{2\leqslant \ell \leqslant n}\sum _{i=1}^{\ell -1} \exp \bigl (-tc^2b^{-\ell }\bigr ) \leqslant n \sum _{2\leqslant \ell \leqslant n} \exp \bigl (- c^2b^{n-\ell }\bigr ) \\&=O(n). \end{aligned} \end{aligned}$$

Moreover, the same argument shows that the expected number of edges *ij* in $\tilde{G}_t$ with $i+j>n+n^{0.1}$ and the number of non-edges *ij* with $i+j<n-n^{0.1}$ both are $O(n b^{-n^{0.1}})$; hence the Borel–Cantelli lemma shows that a.s for every large *n* and every $t\in [b^n,b^{n+1}]$, $\tilde{G}_t$ contains every edge with $i+j< n-n^{0.1}$ and no edge with $i+j> n+n^{0.1}$; a consequence, we also have $[n-n^{0.1}-1]\subseteq V(\tilde{G}_t)\subseteq [n+n^{0.1}]$. It follows that if $H_n$ is the graph with vertex set $\{1,\dots ,n\}$ and edge set $\{ij:i+j\leqslant n\}$, then a.s the cutdistance $\delta _{\square }(\tilde{G}_t,H_n)=o(1)$, when $b^n\leqslant t\leqslant b^{n+1}$. As ${n\rightarrow \infty }$, $H_n\rightarrow \Gamma _{\mathsf {half}}$, the graph limit defined by the graphon $W(x,y)=\varvec{1}_{\{x+y\leqslant 1\}}$ on $[0,1]$ (known as the “half-graphon”). Consequently, $\tilde{G}_{t}\rightarrow \Gamma _{\mathsf {half}}$ a.s as ${t\rightarrow \infty }$. By Proposition 4.9, $G_m\rightarrow \Gamma _{\mathsf {half}}$ a.s as ${m\rightarrow \infty }$.

### Example 8.4

Example 8.3 can be generalized without difficulty. Consider, for example, a rank 1 case $\mu _{ij}=q_iq_j$ with

8.7 $$\begin{aligned} q_i=\exp \bigl (-ci+O(i^{1-\varepsilon })\bigr ) \end{aligned}$$

for some constants $c>0$ and $\varepsilon >0$. Arguing as in Lemma 8.3 we see that a.s , for every large *n* and all $t\in [e^{cn},e^{c(n+1)}]$, $\tilde{G}_t$ contains all edges *ij* with $i+j<n-n^{1-\varepsilon /2}$ and no edges *ij* with $i+j>n+n^{1-\varepsilon /2}$. Consequently, a.s , $\delta _{\square }(\tilde{G}_t,H_n)=o(1)$ and thus $\tilde{G}_t\rightarrow \Gamma _{\mathsf {half}}$ as ${t\rightarrow \infty }$and $G_m\rightarrow \Gamma _{\mathsf {half}}$ as ${m\rightarrow \infty }$.

### Example 8.5

Consider the simple graphs $\tilde{G}_t$ and $G_m$ given by the Hollywood model in Example 7.8 in the case $\alpha =0$. As shown there, the resulting random graphs are the same as the ones given by the rank 1 model with a random probability distribution $(q_i)_1^\infty$ having the distribution $\mathrm {GEM}(0,\theta )$, where $\theta \in (0,\infty )$ is a parameter.

By a well-known characterization of the $\mathrm {GEM}$ distribution, see [35, Theorem 3.2], this means that

8.8 $$\begin{aligned} (q_1,q_2,\dots ) = \bigl ((1-X_1),X_1(1-X_2),X_1X_2(1-X_3),\dots \bigr ), \end{aligned}$$

where $X_i\sim {\text {Beta}}(\theta ,1)$ are i.i.d. In other words, $q_i=(1-X_i)\prod _{j=1}^{i-1}X_j$, and thus

8.9 $$\begin{aligned} \log ( q_i) = \log (1-X_i) + \sum _{j=1}^{i-1}\log (X_j). \end{aligned}$$

Hence, by the law of iterated logarithm, a.s

8.10 $$\begin{aligned} \log (q_i) = -ci + O\bigl (\sqrt{i\log \log i}\bigr )=-ci+O\bigl (i^{0.6}\bigr ), \end{aligned}$$

where $c:=-{\mathbb {E}}\log (X_1)=1/\theta$. Hence, by conditioning on $(q_i)_1^\infty$, Example 8.4 applies. Consequently, $G_m\rightarrow \Gamma _{\mathsf {half}}$ a.s as ${m\rightarrow \infty }$  for the Hollywood model with $\alpha =0$ and any $\theta >0$.

### Example 8.6

For another generalization of Example 8.3, consider the rank 1 case with $q_i\asymp \exp \bigl (-ci^\gamma \bigr )$ for some $c>0$ and $\gamma >0$. It follows by a similar argument that a.s $G_m\rightarrow W$, where *W* is the graphon $\varvec{1}_{\{x^\gamma +y^\gamma \leqslant 1\}}$ on $[0,1]$.

In Examples 8.1–8.6, $G_m$ converges a.s to some graph limit. There are also many examples, see e.g. Sects. 9–10, for which $G_m$ are sparse, which is equivalent to $G_m\rightarrow \Gamma _0$, the zero graph limit defined by the graphon $W(x,y)=0$. In fact, any graph limit can occur as a limit of $G_m$, at least along a subsequence. Moreover, the following result shows that there exists a “chameleon” example where every graph limit occurs as the limit of some subsequence. (Note that this includes that there is a subsequence converging to the zero graph limit $\Gamma _0$, which means that $e(G_m)=o(v(G_m)^2)$ along this subsequences; hence this example is neither dense nor sparse.)

### Theorem 8.7

There exists a matrix $\mu =(\mu _{ij})$ such that a.s the graphs $G_m$ are dense in the space of graph limits, in the sense that for every graph limit $\Gamma$, there exists a subsequence $G_{m_\ell }$ that converges to $\Gamma$.

### Proof

Let $F_k$, $k\geqslant 1$, be an enumeration of all finite (unlabelled) simple graphs without isolated vertices, each repeated an infinite number of times. Let $v_k:=v(F_k)$ and let $f_k(i,j)$ be the adjacency matrix of $F_k$.

Let $N_0:=1$ and, inductively, $N_k:= k v_k N_{k-1}$ for $k\geqslant 1$. Let also

8.11 $$\begin{aligned} a_k:=\prod _{j=1}^k N_j^{-4}. \end{aligned}$$

Clearly, $N_k\geqslant k!$, $N_k\geqslant N_{k-1}$ and $a_k\leqslant a_{k-1}$. Finally let, for $i\ne j$,

8.12 $$\begin{aligned} \mu _{ij}= a_k f_k\Bigl (\Bigl \lceil \frac{i}{kN_{k-1}}\Bigr \rceil , \Bigl \lceil \frac{j}{kN_{k-1}}\Bigr \rceil \Bigr ), \qquad N_{k-1}< i \vee j \leqslant N_k. \end{aligned}$$

Let $I_k:=[1,N_k]$ and divide $I_k$ into the $v_k$ subintervals $I_{k,\ell }:=[(\ell -1)kN_{k-1}+1,\ell kN_{k-1}]$, $\ell =1,\dots v_k$. Note that (8.12) says that if $i\in I_{k,p}$ and $j\in I_{k,q}$ and not both $i,j\in I_{k-1}$, then $\mu _{ij}=a_k f_k(p,q)$.

Let $t_k:=N_k a_k^{-1}$. If $n>k$, then the expected number of edges *ij* in $\tilde{G}^*_{t_k}$ with $i\vee j\in I_n{\setminus } I_{n-1}$ is at most, using (8.11),

8.13 $$\begin{aligned} t_k\sum _{i\vee j\in I_n{\setminus } I_{n-1}}\mu _{ij}\leqslant t_ka_n N_n^2 \leqslant t_k \frac{a_{n-1}}{N_{n}^{2}} =\frac{a_{n-1}}{a_k}\frac{ N_k}{N_{n}^{2}} \leqslant \frac{1}{N_{n}}\leqslant \frac{1}{n!}. \end{aligned}$$

Hence the probability that $\tilde{G}^*_{t_k}$ contains some edge with endpoint not in $I_k\times I_k$ is at most

8.14 $$\begin{aligned} \sum _{n>k}\frac{1}{n!}\leqslant \frac{1}{k!}, \end{aligned}$$

and by the Borel–Cantelli lemma, a.s this happens for only finitely many *k*.

Similarly, if $(i,j)\in I_k^2{\setminus } I_{k-1}^2$, then $\mu _{ij}\in \{0,a_k\}$, and the probability that there exists some such pair (*i*, *j*) with $\mu _{ij}=a_k$ but $N_{ij}(t_k)=0$ is at most

8.15 $$\begin{aligned} N_k^2 e^{-t_ka_k} =N_k^2 e^{-N_k} =O\bigl (N_k^{-1}\bigr )=O\bigl (k!^{-1}\bigr ). \end{aligned}$$

Consequently, again by the Borel–Cantelli lemma, a.s for every large *k*, there exists no such pair (*i*, *j*).

We have shown that a.s for every large *k*, the simple graph $\tilde{G}_{t_k}$ contains no edge with an endpoint outside $I_k$, and for $(i,j)\in I_k^2{\setminus } I_{k-1}^2$, recalling (8.12), if $i\in I_{k,p}$ and $j\in I_{k,q}$, then there is an edge *ij* if and only if $f_k(p,q)=1$. In particular, since $F_k$ has no isolated vertices, every $i\in I_k$ is the endpoint of some edge in $\tilde{G}_{t_k}$ and thus a vertex, but no $i\notin I_k$ is; in other words, a.s for every large *k*, $V(\tilde{G}_{t_k})=I_k$. It follows that if $F_k^*$ is the blow-up of $F_k$ with every vertex repeated $kN_{k-1}$ times, then a.s for every large *k*, the graphs $\tilde{G}_{t_k}$ and $H^*_k$ have the same vertex set $I_k$ and their adjacency matrices can differ only for $(i,j)\in I_{k-1}^2$. Consequently, using Remark 3.1,

8.16 $$\begin{aligned} \begin{aligned} \delta _{\square }(\tilde{G}_{t_k},F_k)&= \delta _{\square }(\tilde{G}_{t_k},F_k^*) \leqslant \Vert W_{\tilde{G}_{t_k}}-W_{F_k^*}\Vert _{\square } \leqslant \Vert W_{\tilde{G}_{t_k}}-W_{F_k^*}\Vert _{L^1}\\&\leqslant \frac{N_{k-1}^2}{N_k^2} =\frac{1}{(kv_k)^2} \leqslant k^{-2}, \end{aligned} \end{aligned}$$

a.s for all large *k*.

Now, let $\Gamma$ be a graph limit, and let $\ell \geqslant 1$. By graph limit theory (or definition), there exists a sequence of graphs $H_j$ with $v(H_j)\rightarrow \infty$ and $\delta _{\square }(H_j,\Gamma )\rightarrow 0$ as $j\rightarrow \infty$; hence we may take *j* so large that $H:=H_j$ satisfies $v(H)>\ell$ and $\delta _{\square }(H,\Gamma )<1/\ell$. $H$ may have isolated vertices, so we define $H'$ by choosing a vertex $v\in H$ and adding an edge from *v* to any other vertex in $H$. Then at most $v(H)-1$ edges are added, and thus, similarly to (8.16),

8.17 $$\begin{aligned} \delta _{\square }(H',H) \leqslant \Vert W_{H'}-W_{H}\Vert _{\square } \leqslant \Vert W_{H'}-W_{H}\Vert _{L^1} \leqslant \frac{2 v(H)}{v(H)^2} <\frac{2}{\ell }. \end{aligned}$$

Moreover, $H'$ has no isolated vertices, and thus $H'$ occurs infinitely often in the sequence $(F_k)$ above. Consequently, a.s., there exists $k>\ell$ such that (8.16) holds and $F_k=H'$. Then, by (8.16) and (8.17),

8.18 $$\begin{aligned} \delta _{\square }(\tilde{G}_{t_k},\Gamma ) \leqslant \delta _{\square }(\tilde{G}_{t_k},F_k)+\delta _{\square }(H',H)+\delta _{\square }(H,\Gamma )<\frac{1}{k^{2}}+\frac{2}{\ell }+\frac{1}{\ell }<\frac{4}{\ell }. \end{aligned}$$

By Proposition 4.9, this means that if $m_\ell :=N(t_k)$, then $\delta _{\square }(G_{m_\ell },\Gamma )<4/\ell$. Since $\ell$ is arbitrary, this completes the proof. (We may choose $m_\ell$ inductively, and choose *k* above so large that $m_\ell >m_{\ell -1}$.) $\square$

The chameleon example in Theorem 8.7 is theoretically very interesting, but it is hardly useful as a model in applications; since the behaviour of $G_m$ changes so completely with *m*, it is a model of nothing rather than a model of everything.

If we want convergence of the full sequence $G_m$ and not just subsequence convergence as in Theorem 8.7, we do not know whether every graph limit can occur as a limit.

### Problem 8.8

For which graph limits $\Gamma$ does there exist a matrix $(\mu _{ij})$ such that for the corresponding simple random graphs, $G_m\rightarrow \Gamma$?

## Sparse Examples

We gave in the preceding section some dense examples. It seems to be more typical, however, that the graph $G_m$ contains many vertices of small degree (maybe even degree 1), and that the graph is sparse. We give here a few, related, rank 1 examples; see also the following section.

### Example 9.1

Consider a rank 1 example with $q_i\asymp i^{-\gamma }$ for some $\gamma >1$. Then (7.6) yields

9.1 $$\begin{aligned} v(t)\asymp \sum _{i\geqslant 1}\bigl (1\wedge (ti^{-\gamma })\bigr ) =\sum _{i\leqslant t^{1/\gamma }} 1 + t\sum _{i> t^{1/\gamma }} i^{-\gamma } \asymp {\left\{ \begin{array}{ll} t^{1/\gamma },&{}t\geqslant 1, \\ t,&{}t<1. \end{array}\right. } \end{aligned}$$

This yields by (7.7), for $t\geqslant 2$,

9.2 $$\begin{aligned} e(t)\asymp \sum _{i\geqslant 1}v(tq_i) \asymp \sum _{i\geqslant 1}v(ti^{-\gamma }) \asymp \sum _{i\leqslant t^{1/\gamma }} t^{1/\gamma }i^{-1} + \sum _{i> t^{1/\gamma }} ti^{-\gamma } \asymp t^{1/\gamma }\log t. \end{aligned}$$

Hence, using Theorem 6.4, a.s $v(\tilde{G}_t)\asymp t^{1/\gamma }$ and $e(\tilde{G}_t)\asymp t^{1/\gamma }\log t$ as ${t\rightarrow \infty }$, and $v(G_m)\asymp m^{1/\gamma }$ and $e(G_m)\asymp m^{1/\gamma }\log m$ as ${m\rightarrow \infty }$. It follows that the average degree in $G_m$ is $\asymp \log m$.

In this example we may also show that the degree distribution has a power-law; we state this as a theorem. There is no standard precise definition of what is meant by a power-law degree distribution; we may say that a random variable *X* has a power-law distribution with exponent $\tau$ if ${\mathbb {P}}(X>x)\asymp x^{-(\tau -1)}$ as ${x\rightarrow \infty }$, but this does not make sense for the degree distribution of a finite graph, so we must either consider the asymptotic degree distribution, provided one exists, or give uniform estimates for a suitable range of *x*. (See e.g. [18, Sects. 1.4.1 and 1.7] for a discussion of power-laws for degree distributions.) We follow here the second possibility.

For a (finite) graph *G*, let $v_{\geqslant k}(G)$ be the number of vertices of degree at least *k*, and let $\pi _{\geqslant k}(G):=v_{\geqslant k}(G)/v(G)$, the probability that a random vertex has degree $\geqslant k$.

### Theorem 9.2

In Example 9.1, the random graphs $G_m$ have a power-law distribution with exponent 2 in the following sense. There exist positive constants *c* and *C* such that a.s for every large *m*,

9.3 $$\begin{aligned}&\pi _{\geqslant k}(G_m)&\leqslant C/k,&1\leqslant k<\infty ,&\end{aligned}$$

9.4 $$\begin{aligned}&\pi _{\geqslant k}(G_m)&\geqslant c/k,&1\leqslant k\leqslant c v(G_m).&\end{aligned}$$

As usual, the same result holds for $\tilde{G}_t$. Note that the restriction $k\leqslant cv(G_m)$ in (9.4) is necessary, and best possible (up to the value of the constants); we necessarily have $\pi _{\geqslant k}(G)=0$ when $k\geqslant v(G)$. Note also that we have the same exponent $\tau =2$ for every $\gamma >1$.

### Proof

As usual, we prove the results for $\tilde{G}_t$; the results for $G_m$ follow by Proposition 4.9. We then can write (9.3)–(9.4) as $v_{\geqslant k}(\tilde{G}_t)\leqslant C v(\tilde{G}_t)/k$, $k\geqslant 1$, and $v_{\geqslant k}(\tilde{G}_t)\geqslant c v(\tilde{G}_t)/k$, $1\leqslant k\leqslant cv(\tilde{G}_t)$, and by Theorem 6.4 and (9.1), it suffices (and is equivalent) to prove that a.s

9.5 $$\begin{aligned}&v_{\geqslant k}(\tilde{G}_t)&\leqslant C_{1} t^{1/\gamma }/k,&1\leqslant k<\infty ,&\end{aligned}$$

9.6 $$\begin{aligned}&v_{\geqslant k}(\tilde{G}_t)&\geqslant c_{1} t^{1/\gamma }/k,&1\leqslant k\leqslant c_{2} t^{1/\gamma },&\end{aligned}$$

for every large *t*.

Let $I_{ij}$ be the indicator of an edge *ij* in $\tilde{G}_t$; thus $I_{ij}\sim {\text {Be}}\bigl (1-e^{-2tq_iq_j}\bigr )$. Let $D_i:=\sum _{j\ne i}I_{ij}$ be the degree of *i* in the simple graph $\tilde{G}_t$. (The degree is defined as 0 if *i* is not a vertex.)

(i) *The upper bound* (9.5) We fix $t\geqslant 1$ and an integer $k\geqslant 1$; for convenience we often omit them from the notation, but note that many variables below depend on them, while all explicit and implicit constant are independent of *t* and *k*. Let $J_i:=\varvec{1}_{\{D_i\geqslant k\}}$ and $N:=\sum _i J_i=v_{\geqslant k}(\tilde{G}_t)$. Let *A* be a large constant, chosen later, and assume that $k\geqslant A$, let $i_0:=At^{1/\gamma }/k$ and let $N^*:=\sum _{i>i_0}J_i$. Thus $N\leqslant N^*+i_0$. If $i\geqslant i_0$, then using (7.8), (6.31) and (9.1),
  9.7 $$\begin{aligned} {\mathbb {E}}D_i\asymp v(tq_i)\asymp v(ti^{-\gamma }) \leqslant v\bigl (A^{-\gamma }k^\gamma \bigr )\asymp k/A. \end{aligned}$$
  Thus ${\mathbb {E}}D_i \leqslant C_{2} k/A$ for some $C_{2}\geqslant 0$, and choosing $A=\max (14C_{2},4)$, we find that ${\mathbb {E}}D_i \leqslant k/14\leqslant (k-1)/7$. Since $D_i$ is a sum $\sum _j I_{ij}$ of independent Bernoulli variables, a Chernoff bound (see e.g. [26, (2.11) and Theorem 2.8]) yields
  9.8 $$\begin{aligned} {\mathbb {E}}J_i={\mathbb {P}}(D_i\geqslant k)\leqslant e^{-k}, \qquad i\geqslant i_0, \end{aligned}$$
  and also, for later use,
  9.9 $$\begin{aligned} {\mathbb {P}}(D_i\geqslant k-1)\leqslant e^{1-k}, \qquad i\geqslant i_0. \end{aligned}$$
  For $i\geqslant t^{1/\gamma }$ we also have, by (9.7) and (9.1),
  9.10 $$\begin{aligned} {\mathbb {E}}D_i \asymp v(ti^{-\gamma }) \asymp ti^{-\gamma }. \end{aligned}$$
  Let $(x)_r:=x(x-1)\cdots (x-r+1)$, the falling factorial. Since $D_i$ is a sum of independent indicators, it is easily seen that for any positive integer *r*, the factorial moment can be bounded by ${\mathbb {E}}(D_i)_r\leqslant ({\mathbb {E}}D_i)^r$. Hence, by (9.10) and Markov’s inequality, since we assume $k\geqslant A\geqslant 4$,
  9.11 $$\begin{aligned} {\mathbb {E}}J_i = {\mathbb {P}}(D_i\geqslant k) \leqslant \frac{{\mathbb {E}}(D_i)_4}{(k)_4} \leqslant \frac{({\mathbb {E}}D_i)^4}{(k)_4} \leqslant C_{3} \frac{(ti^{-\gamma })^4}{k^4} \leqslant C_{3} \frac{ti^{-\gamma }}{k^4}, \qquad i\geqslant t^{1/\gamma }.\nonumber \\ \end{aligned}$$
  (This also follows from [26, (2.10) and Theorem 2.8]). Summing (9.8) and (9.11), we obtain
  9.12 $$\begin{aligned} {\mathbb {E}}N^*= \sum _{i> i_0} {\mathbb {E}}J_i \leqslant \sum _{i_0< i\leqslant t^{1/\gamma }} e^{-k} + \sum _{i> t^{1/\gamma }} C_{3} ti^{-\gamma }/k^4 \leqslant C_{4} t^{1/\gamma }/k^4. \end{aligned}$$
  For the variance of $N^*$, we note that the indicators $J_i$ are not quite independent, since an edge *ij* influences both $J_i$ and $J_j$, but conditioned on $I_{ij}$, $J_i$ and $J_j$ are independent. Hence, for any distinct *i* and *j*,
  $$\begin{aligned} \begin{aligned} {\mathbb {E}}(J_iJ_j)&={\mathbb {P}}(I_{ij}=1){\mathbb {E}}\bigl (J_iJ_j | I_{ij}=1\bigr ) + {\mathbb {P}}(I_{ij}=0){\mathbb {E}}\bigl (J_iJ_j| I_{ij}=0\bigr ) \\&={\mathbb {P}}(I_{ij}=1){\mathbb {E}}\bigl (J_i| I_{ij}=1\bigr ){\mathbb {E}}\bigl (J_j| I_{ij}=1\bigr )\\&\quad + {\mathbb {P}}(I_{ij}=0){\mathbb {E}}\bigl (J_i| I_{ij}=0\bigr ) {\mathbb {E}}\bigl (J_j| I_{ij}=0\bigr ) \\&\leqslant {\mathbb {P}}(I_{ij}=1){\mathbb {P}}(D_i\geqslant k-1){\mathbb {P}}(D_j\geqslant k-1) + {\mathbb {P}}(I_{ij}=0){\mathbb {E}}{J_i} {\mathbb {E}}{J_j} \end{aligned} \end{aligned}$$
  and thus
  9.13 $$\begin{aligned} {\text {Cov}}(J_i,J_j)\leqslant {\mathbb {P}}(I_{ij}=1){\mathbb {P}}(D_i\geqslant k-1){\mathbb {P}}(D_j\geqslant k-1). \end{aligned}$$
  By (9.13) and (9.9), for $i,j\geqslant i_0$ with $i\ne j$,
  9.14 $$\begin{aligned} {\text {Cov}}(J_i,J_j) \leqslant 2tq_iq_j e^{2(1-k)} \leqslant C_{5} t i^{-\gamma }j^{-\gamma }e^{-2k}. \end{aligned}$$
  Consequently, using also (9.12),
  9.15 $$\begin{aligned} \begin{aligned} {\text {Var}}N^*&=\sum _{i,j> i_0}{\text {Cov}}(J_i,J_j) \leqslant {\mathbb {E}}N^*+ C_{5} te^{-2k} \sum _{i,j> i_0}i^{-\gamma }j^{-\gamma } \\&\leqslant C_{4} t^{1/\gamma }k^{-4} + C_{6} te^{-2k} i_0^{2(1-\gamma )} \leqslant C_{7} t^{1/\gamma }k^{-4}. \end{aligned} \end{aligned}$$
  Hence, by Chebyshev’s inequality,
  9.16 $$\begin{aligned} {\mathbb {P}}\bigl (N^*-{\mathbb {E}}N^*>t^{1/\gamma }/k\bigr ) \leqslant \frac{{\text {Var}}N^*}{(t^{1/\gamma }/k)^2} \leqslant C_{7} t^{-1/\gamma }k^{-2}. \end{aligned}$$
  We have so far kept *t* and *k* fixed. We now sum (9.16) over all $k\geqslant A$ and $t=2^\ell$ for $\ell \in \mathbb N$, and find by the Borel–Cantelli lemma that a.s for every large *t* of this form and every $k\geqslant A$, $N^*-{\mathbb {E}}N^*\leqslant t^{1/\gamma }/k$, and consequently, using also (9.12),
  9.17 $$\begin{aligned} N\leqslant N^*+i_0 \leqslant {\mathbb {E}}N^*+ t^{1/\gamma }/k+i_0 \leqslant C_{8} t^{1/\gamma }/k. \end{aligned}$$
  This is (9.5) for $k\geqslant A$ and $t\in \{2^\ell \}$; since $N$ increases with *t*, (9.5) follows in general (with a different constant), a.s for large *t* and all $k\geqslant A$. For $k<A$, (9.3) and (9.5) follow trivially from $v_{\geqslant k}(\tilde{G}_t)\leqslant v(\tilde{G}_t)$.
(ii) *The lower bound* (9.6) Fix again $t\geqslant 1$ and $k\geqslant 1$, let *B* be a large constant chosen later, and assume that $k\leqslant t^{1/\gamma }/B$. Let *L* be the set of odd integers *i* with $1\leqslant i \leqslant i_1:=B^{-1}t^{1/\gamma }/k$, and let *R* be the set of even integers *j* with $1\leqslant j\leqslant 6k$. By our assumption on *k*, $i_1\geqslant 1$, and thus $|L|=\lfloor (i_1+1)/2\rfloor \geqslant i_1/3$. Note that the indicators $\{I_{ij}\}_{i\in L,\,j\in R}$ are independent. For $i\in L$, let $D_i':=\sum _{j\in R} I_{ij}$ and $J'_i=\varvec{1}_{\{D_i'\geqslant k\}}$. Thus the indicators $\{J_i'\}_{i\in L}$ are independent. Also, let $N':=\sum _{i\in L}J_i'$. Since $J_i' \leqslant J_i$, we have $N'\leqslant \sum _{i\in L} J_i \leqslant \sum _{i\geqslant 1}J_i=N=v_{\geqslant k}(\tilde{G}_t)$.

If $i\in L$ and $j\in R$, then $ij\leqslant 6ki_1=6B^{-1}t^{1/\gamma }$, and thus

9.18 $$\begin{aligned} tq_iq_j \geqslant c_{3} t i^{-\gamma }j^{-\gamma } \geqslant c_{4} B^\gamma . \end{aligned}$$

Choose $B:=c_{4}^{1/\gamma }$; then by (9.18), when $i\in L$ and $j\in R$, $tq_iq_j\geqslant 1$ and thus

9.19 $$\begin{aligned} {\mathbb {P}}(I_{ij}=0) = e^{-2tq_iq_j} \leqslant e^{-2}. \end{aligned}$$

Since $|R|=3k$, it follows that if $i\in L$, then ${\mathbb {E}}D_i' \geqslant 3(1-e^{-2})k>2.5k$, and moreover, by a Chernoff bound (e.g. [26, (2.12)]),

9.20 $$\begin{aligned} {\mathbb {P}}(J_i'=0)= {\mathbb {P}}(D_i'<k) \leqslant e^{- k} \leqslant e^{-1}. \end{aligned}$$

Since the indicators $J_i'$ are independent for $i\in L$, another Chernoff bound shows that

9.21 $$\begin{aligned} {\mathbb {P}}(N'<|L|/2) \leqslant e^{-c_{5} |L|} \leqslant e^{-c_{6} i_1}. \end{aligned}$$

Alternatively, (9.20) and a union bound yield

9.22 $$\begin{aligned} {\mathbb {P}}(N'<|L|/2) \leqslant {\mathbb {P}}(N'<|L|)\leqslant \sum _{i\in L} {\mathbb {P}}(J_i'=0) \leqslant i_1 e^{-k}. \end{aligned}$$

If $1\leqslant k \leqslant t^{1/2\gamma }$, then $i_1\geqslant B^{-1}t^{1/2\gamma }$, and thus (9.21) yields ${\mathbb {P}}(N'<|L|/2) \leqslant e^{-c_{7} t^{1/2\gamma }}$. If $t^{1/2\gamma }< k\leqslant t^{1/\gamma }/B$, then (9.22) yields ${\mathbb {P}}(N'<|L|/2) \leqslant i_1 e^{-c_{8} t^{1/2\gamma }}\leqslant C_{9} e^{-c_{9} t^{1/2\gamma }}$. Consequently, for every $k\leqslant t^{1/\gamma }/B$,

9.23 $$\begin{aligned} {\mathbb {P}}(N< |L|/2)\leqslant {\mathbb {P}}(N'<|L|/2) \leqslant C_{10} e^{-c_{10} t^{1/2\gamma }}. \end{aligned}$$

We have kept *k* and *t* fixed, but we now sum (9.23) over all $k\leqslant t^{1/\gamma }/B$ and $t=2^\ell$ for some $\ell \in \mathbb N_0$. It follows by the Borel–Cantelli lemma that a.s for every large *t* of this form and every $k\leqslant t^{1/\gamma }/B$, $N\geqslant |L|/2\geqslant i_1/6 \geqslant c_{11} t^{1/\gamma }/k$. This proves (9.6) for *t* of the form $2^\ell$, and again the general case follows since *N* is monotone in *t*. $\square$

Furthermore, assuming $q_i\sim ci^{-\gamma }$ in Example 9.1, we can show that $\tilde{G}_t$ and $G_m$ converge a.s to a graphon of the type defined by [39] and mentioned in Sect. 5.1; these graphons are measurable functions $W:\mathbb R_+^2\rightarrow [0,1]$, such that the random graphs *G*(*t*, *W*) defined in (5.5) are a.s finite. (See [39] for precise conditions; see also [9, 3] for related versions.) Recall that the standard graphons discussed in Sect. 3 are useful for dense graphs, but not for sparse graphs as here; the more general graphons in [39] are intended for sparse graphs.

Veitch and Roy [40] defined two notions $\rightarrow _{\mathsf {GP}}$ and $\rightarrow _{\mathsf {GS}}$ of convergence for such general graphons on $\mathbb R_+$ (and the even more general graphexes defined in [39]) based on convergence in distribution of the corresponding random graphs *G*(*t*, *W*). We can define $W_n\rightarrow _{\mathsf {GP}}W$ as meaning $G(r,W_n)\overset{\mathrm {d}}{\longrightarrow }G(r,W)$ for each fixed $r<\infty$, see further [40, 24].

Furthermore, the random graphs *G*(*r*, *W*) are naturally coupled for different *r* and form an increasing graph process $(G(r,W))_{r\geqslant 0}$. Let $(G_{\tau _k}(W))_k$ be the sequence of different graphs that occur among *G*(*r*, *W*) for $r\geqslant 0$. Then $W_n\rightarrow _{\mathsf {GS}}W$ if $(G_{\tau _k}(W_n))_k\overset{\mathrm {d}}{\longrightarrow }(G_{\tau _k}(W))_k$; again see further [40, 24].

Recall that for a finite graph *G*, we defined a corresponding graphon $W_G$ in Sect. 3. In the context of graphons on $\mathbb R_+$, [40] define for every $s>0$ a modification $W_{G,s}$, called the *dilated empirical graphon*, as follows. We may assume that *G* has vertices labelled $1,\dots ,v(G)$; then $W_G(i,j):=\varvec{1}_{\{i\sim j\}}$ for $i,j\leqslant v(G)$; we extend this by $W_G(i,j):=0$ when $i\vee j>v(G)$. Then, for every $s>0$, let the dilated graphon $W_{G,s}$ be the function $\mathbb R_+^2\rightarrow \{0,1\}$ given by $W_{G,s}(x,y):=W_G(\lceil sx\rceil ,\lceil sy\rceil )$. Hence, every vertex in *G* corresponds to an interval of length 1 / *s* in the domain of $W_{G,s}$.

If $G_n$ is a sequence of graphs and *W* a graphon, then $G_n\rightarrow _{\mathsf {GS}}W$ means that $W_{G_n}\rightarrow _{\mathsf {GS}}W$; furthermore, the convergence $\rightarrow _{\mathsf {GS}}$ is insensitive to dilations, so $G_n\rightarrow _{\mathsf {GS}}W$ is equivalent to $W_{G_n,s_n}\rightarrow _{\mathsf {GS}}W$ for any sequence $s_n>0$.

### Remark 9.3

We have in Sect. 5.1 given the version of *G*(*r*, *W*) without loops; more generally, one can allow $i=j$ in (5.4) and thus allow loops. The loopless case considered here then is obtained by imposing $W(x,x)=0$ for $x>0$. Hence, for the version with loops, Theorem 9.4 below still holds, provided we redefine *W* to be 0 on the diagonal.

### Theorem 9.4

In Example 9.1, assume that $q_i\sim c i^{-\gamma }$ as $i\rightarrow \infty$, with $c>0$. Then the dilated empirical graphon $W_{\tilde{G}_t,t^{1/2\gamma }}\rightarrow _{\mathsf {GP}}W$ a.s as ${t\rightarrow \infty }$, where *W* is the graphon $W(x,y)=1-\exp \bigl (-2c^2 x^{-\gamma }y^{-\gamma }\bigr )$ on $\mathbb R_+^2$.

As a consequence, $\tilde{G}_t\rightarrow _{\mathsf {GS}}W$ a.s as ${t\rightarrow \infty }$.

Note that $W(x,y)\geqslant 1-\exp (-2c^2)>0$ when $xy\leqslant 1$, and thus $\int W=\infty$.

We prove first two lemmas.

### Lemma 9.5

Let $(Z_{kl})_{k,l}$ be an array of i.i.d. random variables. Furthermore, let $x_1,\dots ,x_n>0$ be distinct and let *X* be a random variable, independent of the array $(Z_{kl})_{k,l}$, with $X\sim U(a,b)$ where $0<a<b<\infty$. Then,

9.24 $$\begin{aligned} {\mathcal L}\bigl ((Z_{\lceil tx_i\rceil ,\lceil tX\rceil })_{i=1}^n| (Z_{kl})_{k,l}\bigr ) \overset{\mathrm {a.s.}}{\longrightarrow }{\mathcal L}\bigl ((Z_{i,n+1})_{i=1}^n\bigr ) \end{aligned}$$

as $${t\rightarrow \infty }$$.

In other words, conditionally on $(Z_{kl})_{k,l}$ and for a.e.every realization of $(Z_{kl})_{k,l}$, the random vector $(Z_{\lceil tx_i\rceil ,\lceil tX\rceil })_{i=1}^n$ converges in distribution to $(Z'_{i,n+1})_{i=1}^n$, where $(Z'_{kl})_{k,l}$ is an independent copy of $(Z_{kl})_{k,l}$.

### Proof

It suffices to prove that for every fixed rational $z_1,\dots ,z_n$,

9.25 $$\begin{aligned} {\mathbb {P}}\bigl (Z_{\lceil tx_i\rceil ,\lceil tX\rceil }\leqslant z_i, 1\leqslant i\leqslant n| (Z_{kl})_{k,l}\bigr ) \overset{\mathrm {a.s.}}{\longrightarrow }\pi :={\mathbb {P}}\bigl (Z_{i,n+1}\leqslant z_i, 1\leqslant i\leqslant n\bigr ), \end{aligned}$$

where

9.26 $$\begin{aligned} \pi =\prod _{i=1}^n{\mathbb {P}}\bigl (Z_{i,n+1}\leqslant z_i\bigr )=\prod _{i=1}^n{\mathbb {P}}(Z_{11}\leqslant z_i). \end{aligned}$$

Let further $I_{k,l,i}:=\varvec{1}_{\{Z_{kl}\leqslant z_i\}}$ and $J_l:=\prod _{i=1}^nI_{\lceil tx_i\rceil ,l,i}$. Then, with the error term coming from edge effects,

9.27 $$\begin{aligned} \begin{aligned}&P_t:= {\mathbb {P}}\Bigl (Z_{\lceil tx_i\rceil ,\lceil tX\rceil }\leqslant z_i, 1\leqslant i\leqslant n| (Z_{kl})_{k,l}\Bigr ) ={\mathbb {E}}\Bigl (\prod _{i=1}^nI_{\lceil tx_i\rceil ,\lceil tX\rceil ,i}| (Z_{kl})_{k,l}\Bigr ) \\&\qquad ={\mathbb {E}}\bigl (J_{\lceil tX\rceil }| (Z_{kl})_{k,l}\bigr ) =\frac{1}{(b-a)t} \sum _{ta<l\leqslant tb} J_l+o(1). \end{aligned} \end{aligned}$$

If *t* is sufficiently large, then $\lceil tx_1\rceil ,\dots ,\lceil tx_n\rceil$ are distinct, and then, see (9.26),

9.28 $$\begin{aligned} {\mathbb {E}}J_l=\prod _{i=1}^n{\mathbb {E}}I_{\lceil tx_i\rceil ,l,i} =\prod _{i=1}^n{\mathbb {P}}\bigl (Z_{\lceil tx_i\rceil ,l}\leqslant z_i\bigr )=\pi . \end{aligned}$$

Furthermore, then the variables $J_l\sim {\text {Be}}(\pi )$ are i.i.d., so their sum in (9.27) has a binomial distribution, and a Chernoff bound shows that for every $\varepsilon >0$, there is a $c=c(\varepsilon )>0$ such that for large *t*,

9.29 $$\begin{aligned} {\mathbb {P}}\bigl (|P_t-\pi |>\varepsilon \bigr )\leqslant e^{-c t}. \end{aligned}$$

This shows that $P_t$ converges to $\pi$ in probability as ${t\rightarrow \infty }$. In order to show convergence a.s, we note that if $0<t<u$, and $t(b-a)>1$, then (for fixed *a* and *b*) ${\mathbb {P}}(\lceil tX\rceil \ne \lceil uX\rceil )=O(u-t)$, and consequently, for some $C>0$,

9.30 $$\begin{aligned} | P_t-P_u|\leqslant {\mathbb {P}}(\lceil tX\rceil \ne \lceil uX\rceil )\leqslant C(u-t). \end{aligned}$$

Let $\varepsilon >0$, let $N:=\lceil C/\varepsilon \rceil$ and let $t_n:=n/N$. By (9.29) and the Borel–Cantelli lemma, a.s $|P_{t_n}-\pi |\leqslant \varepsilon$ for all large *n*. Furthermore, if *n* is large and $t_n\leqslant t\leqslant t_{n+1}$, then (9.30) implies $|P_t-P_{t_n}|\leqslant \varepsilon$, and thus $|P_t-\pi |\leqslant 2\varepsilon$. Consequently, a.s , $|P_t-\pi |\leqslant 2\varepsilon$ for every large *t*. Since $\varepsilon$ is arbitrary, this proves (9.25) and thus the lemma. $\square$

### Lemma 9.6

Let $(Z_{kl})_{k,l}$ be an array of i.i.d. random variables, and let $(X_1,\dots ,X_n)$ be a random vector in $\mathbb R_+^n$ with an absolutely continuous distribution, independent of the array $(Z_{kl})_{k,l}$. Then,

9.31 $$\begin{aligned} {\mathcal L}\bigl ((Z_{\lceil tX_i\rceil ,\lceil tX_j\rceil })_{1\leqslant i<j\leqslant n}| (Z_{kl})_{k,l}\bigr ) \overset{\mathrm {a.s.}}{\longrightarrow }{\mathcal L}\bigl ((Z_{i,j})_{1\leqslant i<j\leqslant n}\bigr ) \end{aligned}$$

as $${t\rightarrow \infty }$$.

### Proof

Step 1 Assume first that $X_1,\dots ,X_n$ are independent with $X_i\sim U(I_i)$ for some intervals $I_1,\dots ,I_n$. In this case we prove (9.31) by induction on *n*, so we may assume that
  9.32 $$\begin{aligned} {\mathcal L}\bigl ((Z_{\lceil tX_i\rceil ,\lceil tX_j\rceil })_{1\leqslant i<j\leqslant n-1}| (Z_{kl})_{k,l}\bigr ) \overset{\mathrm {a.s.}}{\longrightarrow }{\mathcal L}\bigl ((Z_{i,j})_{1\leqslant i<j\leqslant n-1}\bigr ). \end{aligned}$$
  Furthermore, by Lemma 9.5 and conditioning on $X_1,\dots ,X_{n-1}$,
  9.33 $$\begin{aligned} {\mathcal L}\bigl ((Z_{\lceil tX_i\rceil ,\lceil tX_n\rceil })_{1\leqslant i\leqslant n-1}| (Z_{kl})_{k,l}, X_1,\dots ,X_{n-1}\bigr ) \overset{\mathrm {a.s.}}{\longrightarrow }{\mathcal L}\bigl ((Z_{i,n})_{1\leqslant i\leqslant n-1}\bigr ). \end{aligned}$$
  The result (9.31) follows by (9.32) and (9.33), which shows the induction step and completes the proof of this step.
Step 2 Suppose that there exists a finite family of disjoint intervals $I_k$ such that the density function $f(x_1,\dots ,x_n)$ of $(X_1,\dots , X_n)$ is supported on $\bigl (\bigcup _k I_k\bigr )^n$ and constant on each $\prod _{i=1}^nI_{k_i}$. Then Step 1 shows that for each sequence $k_1,\dots ,k_n$ of indices, (9.31) holds conditioned on $(X_1,\dots ,X_n)\in \prod _{i=1}^nI_{k_i}$. Hence (9.31) holds unconditioned too.
Step 3 The general case. Let $f(x_1,\dots ,x_n)$ be the density function of $(X_1,\dots , X_n)$, and let $\varepsilon >0$. Then there exists a density function $f_0(x_1,\dots ,x_n)$ of the type in Step 2 such that $\int |f-f_0|\,\mathrm {d}x_1\dots \,\mathrm {d}x_n<\varepsilon$. We can interpret $f_0$ as the density function of a random vector $\mathbf {X}^0=(X^0_1,\dots ,X^0_n)$, and we can couple this vector with $\mathbf {X}=(X_1,\dots ,X_n)$ such that ${\mathbb {P}}\bigl (\mathbf {X}\ne \mathbf {X}^0\bigr )<\varepsilon$. Since Step 2 applies to $\mathbf {X}^0$, it follows that
  9.34 $$\begin{aligned} {\mathbb {P}}\bigl (\text {the convergence in (9.31) holds}\bigr ) \geqslant {\mathbb {P}}\bigl ((X,Y)=(X_0,Y_0)\bigr )>1-\varepsilon . \end{aligned}$$
  Since $\varepsilon >0$ is arbitrary, (9.31) follows. $\square$

### Proof of Theorem 9.4

Let $$w_x:=c^{-1}q_{\lceil x\rceil } x^{\gamma }=1+o(1)$$, as $${x\rightarrow \infty }$$.

We can construct $\tilde{G}_t$ for all $t>0$ by taking i.i.d. random variables $Z_{kl}\sim {\text {Exp}}(1)$ and letting there be an edge *kl* in $\tilde{G}_t$ if $2tq_kq_l \geqslant Z_{kl}$, for every pair (*k*, *l*) with $k<l$.

Let $\hat{W}_t:=W_{\tilde{G}_t,t^{1/2\gamma }}$ be the dilated empirical graphon in the statement. Fix $r>0$, and consider the random graph $G(r,\hat{W}_t)$; this is by (5.4)–(5.5) obtained by taking a Poisson process $\{\eta _i\}_i$ on $\mathbb R_+$ with intensity *r* (where we assume $\eta _1<\eta _2<\dots$), and then taking an edge *ij* if and only if $\hat{W}_t(\eta _i,\eta _j)=1$. By the definition of $\hat{W}_t$, this is equivalent to $\tilde{G}_t$ having an edge between $\lceil t^{1/2\gamma }\eta _i\rceil$ and $\lceil t^{1/2\gamma }\eta _j\rceil$, and thus by the construction of $\tilde{G}_t$ to (assuming that *t* is large so that $\lceil t^{1/2\gamma }\eta _i\rceil \ne \lceil t^{1/2\gamma }\eta _j\rceil$)

9.35 $$\begin{aligned} 2 t q_{\lceil t^{1/2\gamma }\eta _i\rceil } q_{\lceil t^{1/2\gamma }\eta _j\rceil } \geqslant Z_{\lceil t^{1/2\gamma }\eta _i\rceil ,\lceil t^{1/2\gamma }\eta _j\rceil } \end{aligned}$$

or, equivalently,

9.36 $$\begin{aligned} 2c^2 \eta _i^{-\gamma }\eta _j^{-\gamma } \geqslant w_{{t^{1/2\gamma }\eta _i}}^{-1}w_{{t^{1/2\gamma }\eta _j}}^{-1}Z_{\lceil t^{1/2\gamma }\eta _i\rceil ,\lceil t^{1/2\gamma }\eta _j\rceil }. \end{aligned}$$

Fix $n<\infty$ and consider the edge indicators $I_{i,j,t}$ in $G(r,\hat{W}_t)$ for $1\leqslant i<j\leqslant n$. Furthermore, fix a large integer *N* and condition $(\eta _1,\dots ,\eta _n)$ on $\lceil N\eta _1\rceil ,\dots ,\lceil N\eta _n\rceil$. By Lemma 9.6, and recalling $w_x=1+o(1)$, the distribution of the right-hand sideof (9.36) converges a.s to independent ${\text {Exp}}(1)$ variables, jointly for $1\leqslant i<j\leqslant n$. Since $I_{i,j,t}$ equals the indicator of (9.36), it follows by first replacing the left-hand sideof (9.36) by upper and lower bounds obtained by rounding each $\eta _i$ down or up to the nearest multiple of 1 / *N*, applying Lemma 9.6 and then letting ${N\rightarrow \infty }$, that

9.37 $$\begin{aligned} \begin{aligned} {\mathcal L}\bigl ((I_{i,j,t})_{1\leqslant i<j\leqslant n}| \tilde{G}_t\bigr ) \rightarrow {\mathcal L}\bigl (\bigl (\varvec{1}\bigl \{2c^2\eta _i^{-\gamma }\eta _j^{-\gamma }\geqslant Z_{ij}\bigr \}\bigr )_{1\leqslant i<j\leqslant n}\bigr ). \end{aligned} \end{aligned}$$

Here, conditioned on $\eta _1,\dots ,\eta _n$, the indicators in the right-hand sideare independent, and have (conditional) expectations

9.38 $$\begin{aligned} {\mathbb {P}}\bigl ( 2c^2\eta _i^{-\gamma }\eta _j^{-\gamma }\geqslant Z_{ij}| \eta _i,\eta _j\bigr ) =1-\exp \bigl (-2c^2\eta _i^{-\gamma }\eta _j^{-\gamma }\bigr ) =W(\eta _i,\eta _j). \end{aligned}$$

This equals the (conditional) probability of an edge *ij* in *G*(*r*, *W*). Consequently, if $I_{i,j}$ is the indicator of an edge *ij* in *G*(*r*, *W*), (9.37) shows that a.s , as ${t\rightarrow \infty }$,

9.39 $$\begin{aligned} \bigl ((I_{i,j,t})_{1\leqslant i<j\leqslant n}| \tilde{G}_t\bigr ) \overset{\mathrm {d}}{\longrightarrow }{(I_{i,j})_{1\leqslant i<j\leqslant n}} \end{aligned}$$

This shows the desired convergence $G(r,\hat{W}_t)\overset{\mathrm {d}}{\longrightarrow }G(r,W)$, provided we restrict the graphs to a fixed finite set of vertices.

To extend this to the infinite number of potential vertices, we need a tightness argument. (Unfortunately, we did not find a really simple argument.) Let $a,b>0$, and let $V_{a,b,t}$ denote the number of edges in $G(r,\hat{W}_t)$ with endpoints labelled $\eta _i,\eta _j$ with $\eta _i\in (a,2a]$ and $\eta _j\in (b,2b]$. Then, cf. (9.35),

9.40 $$\begin{aligned} {\mathbb {E}}\bigl (V_{a,b,t}| (Z_{kl})_{k,l}\bigr ) \leqslant \sum _{ k=\lceil at^{1/2\gamma }\rceil }^{\lceil 2at^{1/2\gamma }\rceil }\sum _{l=\lceil bt^{1/2\gamma }\rceil }^{\lceil 2bt^{1/2\gamma }\rceil }r^2t^{-1/\gamma } \varvec{1}\{2tq_kq_l\geqslant Z_{k,l}\}. \end{aligned}$$

For *k*, *l* in the ranges in (9.40), $q_k \leqslant C_{1} a^{-\gamma }t^{-1/2}$ and $q_l \leqslant C_{1} b^{-\gamma }t^{-1/2}$. Define $J_{kl}:=\varvec{1}\{2C_{1}^2a^{-\gamma }b^{-\gamma }\geqslant Z_{k,l}\}$, $S_{m,n}:=\sum _{k\leqslant m, l\leqslant n} J_{kl}$, $\bar{S}_{m,n}:=S_{m,n}/mn$ and $S^*:=\sup _{m,n\geqslant 1} \bar{S}_{m,n}$. Then (9.40) implies, assuming $t\geqslant t_{a,b}:=\max \{a^{-2\gamma },b^{-2\gamma }\}$,

9.41 $$\begin{aligned} \begin{aligned} {\mathbb {E}}\bigl (V_{a,b,t}| (Z_{kl})_{k,l}\bigr )&\leqslant \sum _{ k=\lceil at^{1/2\gamma }\rceil }^{\lceil 2at^{1/2\gamma }\rceil }\sum _{l=\lceil bt^{1/2\gamma }\rceil }^{\lceil 2bt^{1/2\gamma }\rceil } r^2t^{-1/\gamma } J_{kl} \leqslant r^2 t^{-1/\gamma } S_{\lceil 2at^{1/2\gamma }\rceil ,\lceil 2bt^{1/2\gamma }\rceil } \\&\leqslant r^2 t^{-1/\gamma } \lceil 2at^{1/2\gamma }\rceil \lceil 2bt^{1/2\gamma }\rceil S^*\leqslant 9r^2 ab S^*. \end{aligned}\nonumber \\ \end{aligned}$$

Fix $p>1$ with $p<\gamma$. Then by the multi-dimensional version of Doob’s $L^p$ inequality, see [38, Lemma 3], (9.41) implies, for fixed *r*,

9.42 $$\begin{aligned} \begin{aligned} {\mathbb {E}}\sup _{t\geqslant t_{a,b}} {\mathbb {E}}\bigl (V_{a,b,t}| (Z_{kl})_{k,l}\bigr )&\leqslant C_{2} ab {\mathbb {E}}S^*\leqslant C_{2} ab ({\mathbb {E}}(S^*)^p)^{1/p} \leqslant C_{3} ab ({\mathbb {E}}I_{11})^{1/p} \\&\leqslant C_{4} a^{1-\gamma /p}b^{1-\gamma /p}. \end{aligned}\nonumber \\ \end{aligned}$$

Let $\varepsilon >0$, and use (9.42) with $a=2^m\varepsilon$ and $b=2^n\varepsilon$. Then summing over all $(m,n)\in \mathbb Z_+$ with $m\vee n\geqslant N$ implies, using Markov’s inequality,

9.43 $$\begin{aligned} \begin{aligned}&{\mathbb {E}}\sup _{t\geqslant t_{\varepsilon ,\varepsilon }} {\mathbb {P}}\big (\hat{W}_t\,\text {has an edge labelled}\, (x,y) \in [\varepsilon ,\infty )^2{\setminus }[\varepsilon ,2^N\varepsilon ]^2| (Z_{kl})_{k,l}\big )\\&\qquad \leqslant C_{5} 2^{-(\gamma /p-1)N}\varepsilon ^{2(1-\gamma /p)}. \end{aligned}\nonumber \\ \end{aligned}$$

Choosing *N* large enough, this is less than $\varepsilon$. Furthermore, the probability that $\hat{W}_t$ has a vertex labelled $<\varepsilon$ is at most ${\mathbb {P}}(\eta _1<\varepsilon )<\varepsilon$, and we can choose *n* such that ${\mathbb {P}}(\eta _n\leqslant 2^N\varepsilon )<\varepsilon$.

It now follows from (9.39) that for any finite graph *H*,

9.44 $$\begin{aligned} \bigl |{\mathbb {P}}\bigl (G(r,\hat{W}_t)=H| \tilde{G}_t\bigr ) -{\mathbb {P}}(G(r,W)=H)\bigr | \leqslant 3\varepsilon + o(1) \end{aligned}$$

a.s as ${t\rightarrow \infty }$. Since $\varepsilon >0$ is arbitrary, this shows $\bigl (G(r,\hat{W}_t)| \tilde{G}_t\bigr )\overset{\mathrm {d}}{\longrightarrow }G(r,W)$ a.s as ${t\rightarrow \infty }$, for every fixed $r<\infty$, which is the same as $\hat{W}_t\rightarrow _{\mathsf {GP}}W$.

Finally, we note that $\hat{W}_t\rightarrow _{\mathsf {GP}}W$ implies $\hat{W}_t\rightarrow _{\mathsf {GS}}W$, see [40, 24], and that $\rightarrow _{\mathsf {GS}}$ is not affected by dilations of the graphons; hence a.s also $W_{\tilde{G}_t}\rightarrow _{\mathsf {GS}}W$, i.e., $\tilde{G}_t\rightarrow _{\mathsf {GS}}W$. $\square$

### Example 9.7

Consider the simple graphs $\tilde{G}_t$ and $G_m$ given by the Hollywood model in Example 7.8 in the case $0<\alpha <1$. As shown there, the resulting random graphs are the same as the ones given by the rank 1 model with a random probability distribution $(q_i)_1^\infty$ having the distribution $\mathrm {PD}(\alpha ,\theta )$, where $\theta >-\alpha$ is the second parameter. This implies that a.s $q_i\sim Zi^{-1/\alpha }$ for some (random) $Z>0$, see [35, Theorem 3.13]. Consequently, Example 9.1 applies with $\gamma =1/\alpha$ (after conditioning on $(q_i)$). In particular, a.s $v(G_m)\asymp m^\alpha$ and $e(G_m)\asymp m^\alpha \log m$ as ${m\rightarrow \infty }$.

Moreover, $G_m$ has a.s a power-law degree distribution with exponent $\tau =2$ in the sense of Theorem 9.2.

Furthermore, Theorem 9.4 shows that $G_m\rightarrow _{\mathsf {GS}}W$ a.s as ${m\rightarrow \infty }$  and that the dilated empirical graphon converges a.s in the sense $W_{\tilde{G}_t,t^{\alpha /2}}\rightarrow _{\mathsf {GP}}W$, where *W* is the random graphon $W(x,y)=1-\exp \bigl (-2Z^2x^{-1/\alpha }y^{-1/\alpha }\bigr )$ on $\mathbb R_+$.

### Problem 9.8

In the simple graph Hollywood model with $0<\alpha <1$ as in Example 9.7, does the degree distribution of $G_m$ converge (a.s , or at least in probability) as ${m\rightarrow \infty }$? If so, what is the asymptotic distribution? Is it random or deterministic?

## Extremely Sparse Examples

We can obtain extremely sparse examples in several ways.

First, Theorem 6.10 shows that any example including dust or attached stars is extremely sparse.

Another way to obtain extremely sparse graphs is to force the degrees to be bounded, as follows.

### Example 10.1

Let $\mu =(\mu _{ij})_{i,j=1}^\infty$ be a symmetric non-negative matrix with $0<\Vert \mu \Vert <\infty$ and assume that each row contains at most *d* non-zero entries, for some $d<\infty$. (For example, let $\mu$ be a band matrix, with $\mu _{ij}=0$ unless $0<|i-j|\leqslant d/2$.)

Since an edge *ij* can exist only when $\mu _{ij}>0$, it follows that every vertex in $G_m$ has degree at most *d*. Hence the sequence $G_m$ has bounded degree, and in particular $G_m$ is sparse; to be more precise we have

10.1 $$\begin{aligned} v(G_m)\leqslant 2e(G_m) \leqslant d v(G_m). \end{aligned}$$

Less obviously, it is also possible to obtain extremely sparse graphs in the rank 1 case, with a sequence $q_i$ that decreases very slowly (remember that $\sum _i q_i=1$ by assumption). We give one such example.

### Example 10.2

Consider the rank 1 case (Sect. 7.1) with $q_i=c/(i\log ^2i)$ for $i\geqslant 2$, where *c* is the appropriate normalization constant. (Any $(q_i)$ with $q_i\asymp 1/(i\log ^2i)$ would yield the same results below.) Recall that, by comparison with an integral, $\sum _{i\geqslant k} 1/(i\log ^2i)\sim 1/\log k$ as $k\rightarrow \infty$.

We will see that (in a sense made precise below) almost all edges belong to stars, and that, moreover, most edges and vertices belong to a small (finite) number of stars; in particular, most vertices have degree 1.

For large *t*, let $\ell (t):=\lfloor t/\log ^2t\rfloor$. Then $\ell (t)\log ^2\ell (t)\sim t$, and thus, using (7.6),

10.2 $$\begin{aligned} v(t)\asymp \sum _{i=1}^\infty \bigl ((q_it)\wedge 1\bigr ) \asymp \sum _{i\leqslant \ell (t)}1+\sum _{i>\ell (t)}\frac{t}{i\log ^2i} \asymp \ell (t)+\frac{t}{\log \ell (t)} \asymp \frac{t}{\log t}. \end{aligned}$$

The expected number of edges is by (7.5),

10.3 $$\begin{aligned} e(t)\asymp \sum _{i\ne j} \bigl ((q_iq_jt)\wedge 1\bigr ) \asymp \sum _{i\ne j} \frac{t}{i\log ^2(i+1)\,j\log ^2(j+1)}\wedge 1 \end{aligned}$$

We split the sum in (10.3) into three (overlapping) parts. The case $j\geqslant t^{0.4}$ yields at most

10.4 $$\begin{aligned} \sum _{i\geqslant 1}\sum _{j\geqslant t^{0.4}} \frac{t}{i\log ^2(i+1)\,j\log ^2(j+1)} \leqslant C_{1} \frac{t}{\log t}. \end{aligned}$$

The case $i\geqslant t^{0.4}$ yields the same, and finally the case $i,j<t^{0.4}$ yields at most

10.5 $$\begin{aligned} \sum _{i<t^{0.4}}\sum _{j< t^{0.4}}1 <t^{0.8}=o\Bigl (\frac{t}{\log t}\Bigr ). \end{aligned}$$

By (10.3)–(10.5), and the lower bound (6.24), we find

10.6 $$\begin{aligned} e(t) \asymp \frac{t}{\log t} \asymp v(t). \end{aligned}$$

Thus Theorem 6.4 yields $e(\tilde{G}_t)\asymp v(\tilde{G}_t)$ and $e(G_m)\asymp v(G_m)$ a.s. In other words, $G_m$ is extremely sparse.

We can be more precise. Recall that $N_i(t)$ is the degree of vertex *i* in $\tilde{G}^*_t$; by (6.3) $N_i(t)\sim {\text {Po}}(\mu _i t)$. Consequently, using also (7.2),

10.7 $$\begin{aligned} {\mathbb {E}}\bigl (N_i(t)\varvec{1}_{\{N_i(t)>1\}}\bigr ) \leqslant {\mathbb {E}}\bigl (N_i(t)(N_i(t)-1)\bigr ) =(\mu _it)^2\leqslant 4q_i^2t^2. \end{aligned}$$

Summing over $i>t/\log ^2t$ we obtain

10.8 $$\begin{aligned} \sum _{i>t/\log ^2t} {\mathbb {E}}\bigl (N_i(t)\varvec{1}_{\{N_i(t)>1\}}\bigr ) \leqslant C_{2} \sum _{i>t/\log ^2 t} \frac{t^2}{i^2\log ^4 i} =O\Bigl (\frac{t}{\log ^2t}\Bigr ). \end{aligned}$$

Hence the expected number of edges that have one endpoint in $(t/\log ^2t,\infty )$ and that endpoint is not isolated is $O(t/\log ^2t)$. Moreover, the expected number of edges with both endpoints in $[1,t/\log ^2t]$ is at most

10.9 $$\begin{aligned} \sum _{i<j\leqslant t/\log ^2t}2((tq_iq_j)\wedge 1) \leqslant t^{0.8} + \sum _{t^{0.4}<j\leqslant t/\log ^2t}\sum _{i<j}2((tq_iq_j)\wedge 1) \end{aligned}$$

where the last sum is at most a constant times, cf. (10.2),10.10It follows by (10.8), (10.9) and (10.10) that, in $\tilde{G}^*_t$ and thus in $\tilde{G}_t$, all but $o_{\mathrm p}(t/\log t)$ edges have one endpoint isolated. If the number of such edges in $\tilde{G}_t$ is $e'(\tilde{G}_t)$, then thus the total number of edges is $e(\tilde{G}_t)=e'(\tilde{G}_t)+O_{\mathrm p}(t/\log ^2t)$, and since each edge has at most two endpoints, the number of vertices is at least $e'(\tilde{G}_t)$ and at most $2e'(\tilde{G}_t)+O_{\mathrm p}(t/\log ^2t)$. Moreover, it is easily seen that the expected number of edges with both endpoints in $(t/\log ^2t,\infty )$ is $O(t/\log ^2t)$, and it follows that, in fact, $v(\tilde{G}_t)=e'(\tilde{G}_t)+O_{\mathrm p}(t/\log ^2t)$; we omit the details. Consequently, using also (6.26) and (10.6), it follows that $e(\tilde{G}_t)/v(\tilde{G}_t)\overset{\mathrm {p}}{\longrightarrow }1$ as ${t\rightarrow \infty }$. Moreover, we see also that almost all edges belong to stars. (These are not attached stars in the sense of Sect. 5, since our example contains no attached stars, but they have a similar effect on the graph.) As a consequence, at least in probability, most vertices have degree 1, so the asymptotic degree distribution is concentrated at 1.

Furthermore, a large fraction of the edges (and thus vertices) belong to a finite number of such stars. To be precise, let $\varepsilon >0$; then there exists an integer $K=K(\varepsilon )<\infty$ such that summing over $i>K$ only in (10.4) yields $<\varepsilon t/\log t$, which together with (10.5) and (10.8)–(10.10) shows that the expected number of edges that are not in a star with centre at *i* for some $i\leqslant K$ is $O\bigl (\varepsilon t/\log t\bigr )=O\bigl (\varepsilon e(t)\bigr )$.

Since a.s $G_m\subseteq \tilde{G}_{2m}$ for all large *m*, the same results follow also for $G_m$.

Unfortunately, these properties make the random graphs in this example rather uninteresting for applications.

## Conclusions

For the multigraph version, the examples in Sect. 7 seem very interesting, but perhaps a bit special. We do not know whether they are typical for a large class of interesting examples or not.

For the simple graph version, the examples above show a great variety of different behaviour. Nevertheless, the results are somewhat disappoining for applications; the relations between the intensity matrix $(\mu _{ij})$ and properties of the random graphs $G_m$ such as edge density and degree distribution are far from obvious, and it is not clear how one can choose the intensity matrix to obtain desired properties; for example, we do not know any example of a power-law degree distribution with an exponent $\tau \ne 2$.

Consequently, for both versions, it seems desirable to study more examples, as well as to find more general theorems.

The present paper is only a first step (or rather second step, after [7, 8, 11, 12]), of the investigation of these random graphs, and it seems too early to tell whether they will be useful as random graph models for various applications or not.

## Footnotes

1 Usually I keep a distiction between graphs and graphons (and graph limits); this is easiest done by identifying a graph *G* with the pair $(W_G,1/v(G))\in \mathcal W\times [0,1]$ (and a graphon *W* with (*W*, 0)), see also [14]. In the present paper, this point of view is not needed.
